# Identification of Common Driver Gene Modules and Associations between Cancers through Integrated Network Analysis

**DOI:** 10.1002/gch2.202100006

**Published:** 2021-06-19

**Authors:** Bo Gao, Yue Zhao, Yonghang Gao, Guojun Li, Ling‐Yun Wu

**Affiliations:** ^1^ IAM MADIS NCMIS Academy of Mathematics and Systems Science Chinese Academy of Sciences Beijing 100190 China; ^2^ School of Mathematics Shandong University Jinan 250100 China; ^3^ School of Mathematical Sciences University of Chinese Academy of Sciences Beijing 100049 China; ^4^ School of Public Health Capital Medical University Beijing 100069 China; ^5^ Beijing Municipal Key Laboratory of Clinical Epidemiology Beijing 100069 China; ^6^ Research Center for Mathematics and Interdisciplinary Sciences Shandong University Qingdao 266237 China

**Keywords:** driver gene module, gene mutation, mutual exclusivity, network analysis, signaling pathway

## Abstract

High‐throughput biological data has created an unprecedented opportunity for illuminating the mechanisms of tumor emergence and evolution. An important and challenging problem in deciphering cancers is to investigate the commonalities of driver genes and pathways and the associations between cancers. Aiming at this problem, a tool ComCovEx is developed to identify common cancer driver gene modules between two cancers by searching for the candidates in local signaling networks using an exclusivity‐coverage iteration strategy and outputting those with significant coverage and exclusivity for both cancers. The associations of the cancer pairs are further evaluated by Fisher's exact test. Being applied to 11 TCGA cancer datasets, ComCovEx identifies 13 significantly associated cancer pairs with plenty of biologically significant common gene modules. The novel results of cancer relationship and common gene modules reveal the relevant pathological basis of different cancer types and provide new clues to diagnosis and drug treatment in associated cancers.

## Graphical abstract

This article presents a network‐based method ComCovEx to predict the common driver gene modules between two cancer types. An exclusivity‐coverage iteration strategy is designed for identifying the gene modules with significant coverage and exclusivity for two cancers. Then the identified gene modules are evaluated by statistical analysis. The associations of cancer pairs are further evaluated by Fisher's exact test.

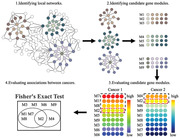

## 1 Introduction

Cancer is a complex disease driven largely by aberrant somatic alterations that accumulate over the lifetime of an individual. High‐throughput sequencing technologies have revolutionized the measurement of somatic alterations in an unprecedented state. Several large‐scale programs, such as The Cancer Genome Atlas (TCGA),^[^ ^1^ ^]^ the International Cancer Genome Consortium (ICGC),^[^ ^2^ ^]^ have generated cancer genomics data for thousands of tumors covering various cancer types. A crucial problem in the analysis and interpretation of the genomic data is to distinguish driver mutations that are functionally related to cancer initiation and development from other passenger ones.^[^ ^3^, ^4^, ^5^ ^]^ However, the complicated heterogeneity of cancer mutations hinders efforts to identify driver mutations and genes with high mutation frequency.^[^ ^6^, ^7^, ^8^ ^]^

It is commonly hypothesized that cancer is caused by pathway defects.^[^ ^9^ ^]^ Evidences suggest that driver mutations typically target a limited number of key pathways.^[^ ^10^, ^11^, ^12^ ^]^ The mutational heterogeneity in cancer is explained by the different combinations of driver mutations in pathways. Several methods have been applied or proposed for the examination of mutations in known pathways or interaction networks.^[^ ^13^, ^14^, ^15^ ^]^ For example, the gene expression analysis tools DAVID,^[^ ^16^, ^17^ ^]^ FaTiGO,^[^ ^18^ ^]^ and GoStat^[^ ^19^ ^]^ can be used to evaluate the significance of overlaps between a list of mutated genes and known gene sets. With a rank list of mutated genes, GSEA^[^ ^20^ ^]^ assesses whether a gene set is enriched by the high‐ranking genes. Unfortunately, an unavoidable difficulty of these methods comes from the incomplete knowledge of known pathways and interaction networks.

An important observation is that driver mutations in a pathway tend to be mutually exclusive, i.e., at most one mutation in the pathway occurs in most cancer samples.^[^ ^21^, ^22^, ^23^, ^24^ ^]^ It is explained that one mutation is sufficient to disturb the pathway and further promotes the development of cancer. De novo methods have been designed to identify combinations of mutations with mutual exclusivity. Miller et al.^[^ ^25^ ^]^ proposed an online‐learning method RME to identify gene sets by considering pairwise exclusivity. Vandin et al.^[^ ^26^ ^]^ designed a combinatorial function Dendrix balancing coverage and mutual exclusivity of a gene set which were optimized by different methods.^[^ ^26^, ^27^, ^28^, ^29^ ^]^ Here, the coverage was defined as the number of cancer samples with mutations in the gene set. Leiserson et al.^[^ ^28^ ^]^ and Zhang et al.^[^ ^30^ ^]^ generalized Dendrix to identify multiple gene sets of high coverage and exclusivity simultaneously. Zhang et al.^[^ ^31^ ^]^ also generalized Dendrix as two mathematical programming models to discover the common and specific driver gene sets of different cancers. However, the Dendrix‐based function tends to be dominated by mutations or genes with large frequencies in real applications.^[^ ^32^, ^33^ ^]^ Li et al.^[^ ^34^ ^]^ further developed a method to identify cancer specific driver gene sets by constructing a specific gene network for each cancer which integrates the coverage and mutual exclusivity. More mutual exclusivity‐based methods can be found.^[^ ^35^ ^]^

Some other methods integrated protein‐protein interaction (PPI) networks to discover driver gene modules.^[^ ^36^, ^37^, ^38^, ^39^ ^]^ A gene module is a set of functionally or topologically related genes in this work. For example, mutated subnetworks with significantly large coverages have been identified by HotNet2.^[^ ^36^ ^]^ MEMo^[^ ^37^ ^]^ and MEMCover^[^ ^38^ ^]^ devoted to identify mutually exclusive modules, but encountered difficulties in computational complexity. Another mutually exclusive module identification method mutex limited the search scope to reduce computational cost.^[^ ^39^ ^]^ DMRG^[^ ^40^ ^]^ incorporated the functional similarity, coverage and mutual exclusivity to detect driver modules with rarely mutated genes in cancers. Recently, we proposed a two‐stage method CovEx to discover gene modules of significantly large coverages and mutual exclusivity by decomposing the problem into a number of smaller problems.^[^ ^33^ ^]^ Considering that the exclusive modules identified by the previous methods may be dominated by a few large coverage genes, we further designed UniCovEx to discover cancer driver gene modules balanced between exclusivity and coverage.^[^ ^41^ ^]^ However, it is still challenging for comparative analysis between different cancer types.

**Figure 1:**
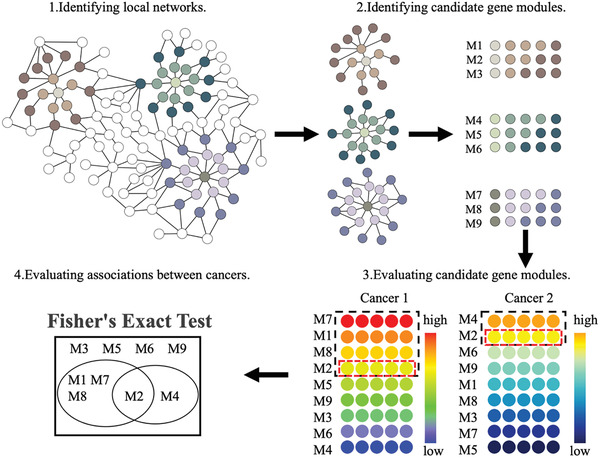
Flowchart of the ComCovEx algorithm. 1) ComCovEx searches for the local networks centered at each considered gene in an influence network derived from a PPI network (see the Experimental Section). 2) ComCovEx identifies the candidate common driver gene modules (such as the modules M1‐M9 in the figure) for the considered cancer pairs in each local network by an exclusivity‐coverage iteration strategy. 3) ComCovEx outputs the predicted common driver gene modules (M2 in the figure) with significantly large coverages and mutually exclusive scores for both cancer types. The color code from low to high corresponds to the low to high significance of a module for a cancer type. The gene modules in the black and red dashline boxes are significant for one cancer type and both cancer types, respectively. 4) ComCovEx conducts association analysis for a cancer pair by Fisher's exact test based on the candidates and the significant gene modules.

In this article, we developed a network‐based method ComCovEx to systematically identify the common driver gene modules between two cancer types (see the Experimental Section). ComCovEx first searches for the local networks centered at each considered gene in an influence network obtained by a random walk model based on a PPI network (step 1 in **Figure** **1**). Then ComCovEx identifies the candidate common driver gene modules in each local network by an exclusivity‐coverage iteration strategy (step 2 in Figure 1), and outputs the predicted common driver gene modules with significantly large coverages and mutually exclusive scores for both cancer types (step 3 in Figure 1). Finally, ComCovEx conducts association analysis for each cancer pair by Fisher's exact test based on the candidates and the output significant modules (step 4 in Figure 1).

When applied to 11 TCGA cancer datasets, ComCovEx identified 13 significantly associated cancer pairs with plenty of biologically significant gene modules. Especially, there are significant association between each cancer pair for the three‐cancer group, BRCA, HNSC, LUSC with the predicted common driver genes of CCND1, ESR1, LCK, NCOA6, PIK3CA, PLK3, STAT3, TP53. The evaluated associations of cancers are also supported by previous experimental studies. Compared to another state‐of‐the‐art method ComMDP,^[^ ^31^ ^]^ ComCovEx identified a number of novel cancer genes and covered more NCG cancer genes which were downloaded from The Network of Cancer Genes 6.0 database (see the Experimental Section). The identified common gene modules significantly overlap with known cancer genes and pathways in databases and literature, and thus providing some important insight to the molecular mechanisms of cancers. The pathway enrichment results may reveal the related pathological basis and provide new clues to diagnosis and drug treatment in related cancers. The association analysis also identified 18 significantly unassociated cancer pairs and suggested that LAML and OV have little association with other cancers indicating the unique pathological mechanisms.

## 2 Results

### 2.1 ComCovEx Identified Significantly Associated Cancer Pairs

**Figure 2:**
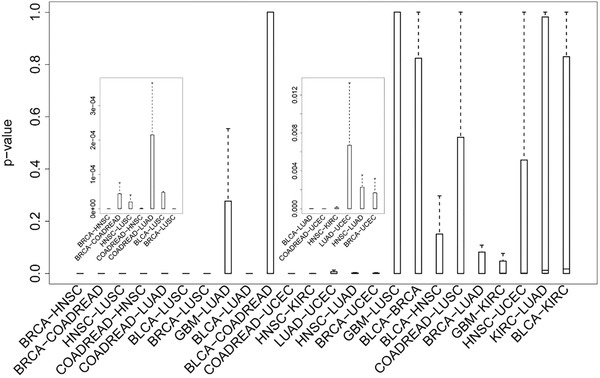
The boxplot of the *p*‐values of the 24 cancer pairs with significant adjusted median (<0.05) *p*‐values. The sequence of the cancer pairs is arranged by the adjusted median *p*‐values in an ascending order. Two small diagrams with different scales have been embedded.

We considered 11 cancer datasets covering 12 cancer types and 3 PPI networks. Totally, 55 cancer pairs were involved in the analysis. For each cancer pair, ComCovEx obtained twelve *p‐*values with Fisher's exact test by considering each of the module sizes ranging from 2 to 5 and each of the three PPI networks. The *p‐*values of the 55 cancer pairs were shown by boxplots (**Figure** **2**; Figure S1, Supporting Information).

**Figure 3:**
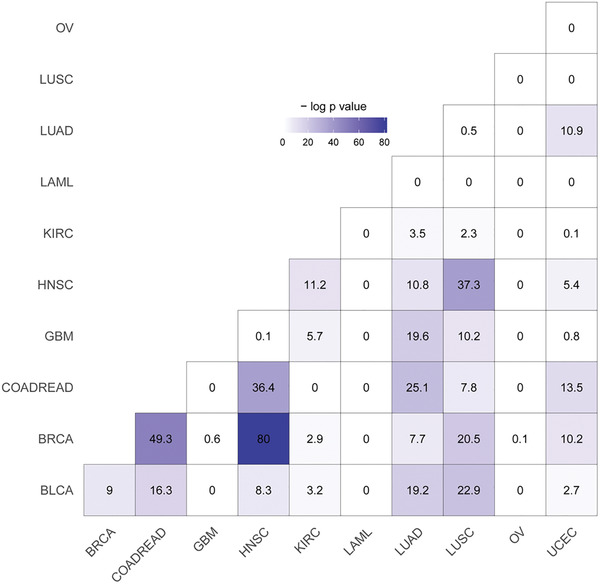
The negative logarithms (base e) of the adjusted Q2 values of all the 55 cancer pairs. Of all cancer pairs, we had 24 cancer pairs with significant adjusted Q2 (<0.05) values. The negative logarithm values are at least 3.0 for the significant cancer pairs in this plot.

**Figure 4:**
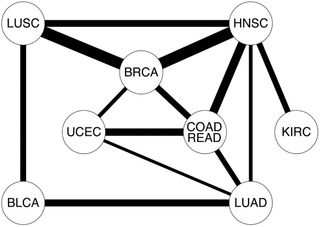
The 13 cancer pairs with significant adjusted Q3 (<0.05) values. The cancer pair with an edge in the graph has significant adjusted Q3 values. The thickness of the edge in the graph is proportional to the negative logarithm of the adjusted Q3 value of the corresponding cancer pair.

The quartiles of the twelve *p*‐values, including the lower quartile (Q1), the middle quartile (Q2) and the upper quartile (Q3), were determined to evaluate the association degree of a cancer pair. Of the 55 cancer pairs, we had 37, 24 and 13 cancer pairs respectively with significant adjusted Q1, Q2, and Q3 (<0.05) values (**Figure** **3**; Figures S2 and S3, Supporting Information). The 13 cancer pairs with significant adjusted Q3 values were identified as significantly associated cancer pairs (**Figure** **4**). On the contrary, the 18 cancer pairs with non‐significant adjusted Q1 values were identified as significantly unassociated cancer pairs.

Of the 13 significantly associated cancer pairs in Figure 4, HNSC was paired with five other cancers. BRCA, COADREAD and LUAD were paired with four other cancers. LUSC and UCEC were paired with three other cancers. Meanwhile, GBM, LAML and OV were paired with no other cancers. In addition, the 18 significantly unassociated cancer pairs included the pair of KIRC and COADREAD, the pairs of LAML pairing with all the other ten cancers and the pairs of OV pairing with all the other ten cancers except for BRCA and UCEC. In fact, the pairs of OV pairing with BRCA and UCEC were not significant for the adjusted Q2 values (0.93 and 1). The results showed that LAML and OV had little association with other cancers, indicating their unique pathogenic mechanisms.

The identified significantly associated cancer pairs are also supported by previous experimental studies. Previous studies have suggested that women with breast cancer were at increased risk of developing long‐term complications from postmastectomy radiotherapy, including lymphedema, brachial plexopathy, pneumonitis, and late cardiac complications.^[^ ^42^, ^43^ ^]^ Postmastectomy radiotherapy for breast cancer increased the risk of lung cancer.^[^ ^44^, ^45^ ^]^ It has been observed that squamous cell lung carcinoma occurred in the irradiated field after radiotherapy for breast cancer.^[^ ^46^ ^]^ Breast cancer radiotherapy and cigarette smoking increased the risk of a subsequent lung carcinoma.^[^ ^43^, ^47^, ^48^ ^]^ Numbers of previous studies have indicated the common biomarkers and potential targets of therapies for both breast cancer and head and neck squamous cell carcinoma.^[^ ^49^, ^50^, ^51^, ^52^, ^53^, ^54^, ^55^ ^]^ The long non‐coding RNA MALAT1 which is a predictive marker for metastasis development in lung cancer was indicated to play an oncogenic role in urothelial carcinoma of the bladder.^[^ ^56^, ^57^ ^]^ Good markers TTF1 and GATA3 for differential diagnosis of primary lung adenocarcinoma (TTF1+ GATA3‐) and metastatic bladder cancer (or breast cancer) (TTF1‐ GATA3+) have been found to be simultaneously expressed in a lung biopsy sample.^[^ ^58^ ^]^ Furthermore, a case was reported that urinary bladder transitional cell carcinoma, metachronous prostate adenocarcinoma and small cell lung carcinoma were diagnosed for the same person within an eighteen‐month period.^[^ ^59^ ^]^ Another case of lung cancer with cavitation was indicated to be caused by metastasis of urothelial carcinoma.^[^ ^60^ ^]^

Our predictions also have a great consistency with a previous computational study which uncovered stratifications among tumors by dividing pan‐cancer patients to subgroups.^[^ ^61^ ^]^ To compare with our predictions, we reanalyzed their micro‐scale pan‐cancer subgroups results (Figure 3A in their paper). For each cancer type, we merged all subtypes and obtained the patient counts distributed on all the nine subgroups. A cancer pair with a PCC (Pearson Correlation Coefficient) value at least 0.80 was regarded as a significantly associated cancer pair. Then we obtained 8 significantly associated cancer pairs with 5 cancer pairs identified as significantly associated by our method (see Table S1 in the Supporting Information). The 5 cancer pairs included all the three significantly associated cancer pairs of UCEC and others, the cancer pair of BRCA and COADREAD and the cancer pair of COADREAD and LUAD. Our method also identified some novel significantly associated cancer pairs such as BRCA and LUSC, since the distinct effects of driver genes and passenger genes were considered.

We obtained interesting observations for the four results by considering the adjusted Q2 or Q3 values of the cancer pairs and different scoring ways (Tables S2–S5, Supporting Information). All the cancers can be divided into five groups. The first group included BRCA and HNSC which always had the highest scores. The second group included COADREAD, LUAD and LUSC which always had high scores just next to the first group. The third group included UCEC and BLCA which always had median scores. The fourth group included KIRC and GBM which always had low scores. The fifth group included OV and LAML which always had the lowest scores. Ideally, the cancers with higher scores may be more likely to be associated with and be more susceptible to other cancers.

### 2.2 ComCovEx Identified the Common Driver Genes and Pathways between Cancers

Although previous studies verified the associations of different cancers, it has not been comprehensively studied for the common driver gene modules and pathways of the associated cancers. For each cancer pair, ComCovEx identified the common driver gene modules for each of the three PPI networks HINT+HI2012, iRefIndex and Multinet, respectively. For all the identified gene modules, we filtered out those genes identified for only one PPI network in our analysis, which would improve the prediction precision. In fact, the precision has been improved for all the 13 significantly associated cancer pairs except for one cancer pair based on the NCG and known cancer genes (Tables S6 and S7, Supporting Information). Especially, the average precisions of the NCG and known cancer genes for the 13 significantly associated cancer pairs have been improved from 47.2% and 30.8% to 57.1% and 42.8%, respectively.

The enrichment analysis with statistical test was conducted by Metascape [http://metascape.org].^[^ ^62^ ^]^ For each given gene list, pathway and process enrichment analysis has been carried out with the following ontology sources: KEGG Pathway, GO Biological Processes, Reactome Gene Sets, Canonical Pathways, CORUM, TRRUST, DisGeNET and PaGenBase. The disease association analysis has been carried out in DisGeNET.^[^ ^63^ ^]^ The hierarchical clustering was performed on the enriched terms. The most statistically significant term within a cluster was chosen to represent the cluster. We selected the default parameters of Metascape in our application.

Identifying the common driver genes and modules between cancers would be helpful for revealing the common pathogenic mechanism and providing new clues to diagnosis and drug treatment in related cancers. To demonstrate the power of ComCovEx for such purpose, we used three significantly associated cancer pairs as examples: the cancer pair of bladder urothelial carcinoma (BLCA) and lung adenocarcinoma (LUAD), the cancer pair of breast invasive carcinoma (BRCA) and head and neck squamous cell carcinoma (HNSC), and the cancer pair of breast invasive carcinoma (BRCA) and lung squamous cell carcinoma (LUSC). We also analyzed some three‐cancer groups within which each cancer pair was significantly associated.

We visualized the induced networks and the Reactome functional interaction (FI) network of the identified driver genes in the filtered modules for the selected three cancer pairs (Figures S4–S6, Supporting Information). Here, the induced network was constructed by the edges existed on at least two of the three influence networks. The Reactome FI network was obtained by the plugin ReactomeFIPlugIn^[^ ^64^ ^]^ in Cytoscape.^[^ ^65^ ^]^ In application, the Reactome FI network version has been selected as 2019. We also applied another Cytoscape plugin GeneMANIA^[^ ^66^, ^67^ ^]^ to visualize the induced networks and the known pathway networks (Figures S7–S9, Supporting Information). Note that the edges in the induced network only indicate the strong association of the corresponding genes rather than the direct interaction in pathways or PPI networks. The identified gene modules have also been compared to those identified by the method ComMDP. For each cancer pair, ComMDP was run for the module size from 2 to 10 (Tables S8–S10, Supporting Information).

#### 2.2.1 Bladder Urothelial Carcinoma (BLCA) and Lung Adenocarcinoma (LUAD)

**Figure 5:**
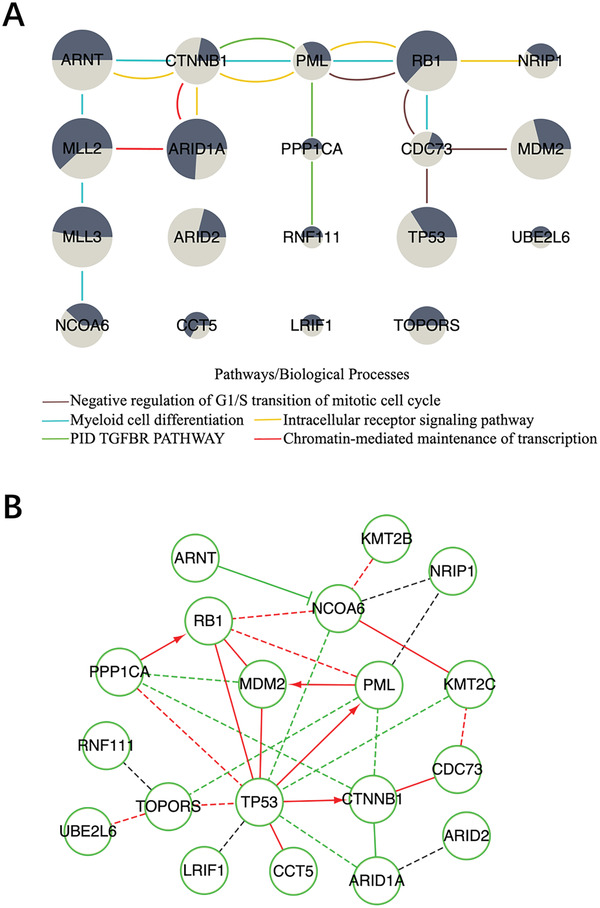
The enrichment analysis and the visualization of the driver gene module identified by ComCovEx for BLCA and LUAD. A) The enriched pathways and biological processes of the gene sets identified by ComCovEx for BLCA and LUAD. The sizes of nodes (genes) are proportional to the coverage of the corresponding genes for both cancers. Here, if the coverage of a gene is more than 15, then the coverage of the gene is regarded as 15 in the plot. Genes connected by the same color edges belong to the same pathway or biological process. The two areas of dark and light colors at each gene are proportional to the coverages of the gene for BLCA and LUAD, respectively. B) The visualization of the induced network and the Reactome FI network of the identified driver genes for BLCA and LUAD. The induced network edges with paths in the Reactome FI network connecting the corresponding genes have been removed. The black dotted edges belong to the induced network and may reflect the possible indirect functional interactions between genes/proteins. Besides the black dotted edges, the other green and red edges are Reactome FI edges obtained directly from the plugin. According to the annotations of the plugin, “→” for activating/catalyzing, “‐|” for inhibition, “‐” for FIs extracted from complexes or inputs, and “‐‐‐” for predicted FIs. Especially, the red edges are both the Reactome FI edges and the induced network edges.

We identified a total of 19 driver genes with 13 NCG cancer genes in the filtered gene modules (**Figure** **5**A). The pathway and process enrichment analysis indicated that the enriched terms were grouped into nine clusters (Figure S10, Supporting Information). The most significant five representative terms included four GO biological processes terms and one Canonical Pathways term, such as myeloid cell differentiation (GO:0030099), negative regulation of G1/S transition of mitotic cell cycle (GO:2000134), intracellular receptor signaling pathway (GO:0030522), chromatin‐mediated maintenance of transcription (GO:0048096) and PID TGFBR PATHWAY (M286).

Of all the 19 predicted driver genes, the most significant five representative terms covered 14 genes with CTNNB1, PML and RB1 belonging to at least three representative terms. All the significant nine representative terms covered 16 genes with 6 NCG cancer genes CTNNB1, PML, RB1, MLL2, ARNT and NRIP1 belonging to at least three representative terms. The 16 genes included 12 NCG cancer genes with exception for CCT5, NCOA6, PPP1CA and TOPORS. Three genes ARID2, LRIF1 and UBE2L6 were not covered by any representative term with only ARID2 being an NCG cancer gene.

Enrichment analysis in DisGeNET indicated that these genes were significantly enriched for liver carcinoma (C2239176), sezary syndrome (C0036920), osteosarcoma (C0029463), adrenal gland neoplasms (C0001624) and transitional cell carcinoma of bladder (C0279680) (Figure S11, Supporting Information). Especially, three genes CTNNB1, RB1 and TP53 were enriched for transitional cell carcinoma of bladder (C0279680). Four genes RB1, TP53, ARID1A and KMT2C were enriched for bladder neoplasm (C0005695).

We found a number of overlaps between the induced network and the Reactome FI network of the identified driver genes (Figure S4, Supporting Information). Considering that edges in the induced network may only indicate the indirect association of the corresponding genes, we further removed the induced network edges with paths in the Reactome FI network connecting the corresponding genes (Figure 5B). We had five induced network edges left, such as the edge connecting ARID1A and ARID2, etc. We believe that the left edges deserve further study for their possible unknown functional interaction.

We run ComMDP for BLCA and LUAD (Table S7, Supporting Information). Combining all the identified gene sets with size from 2 to 10, ComMDP identified 19 cancer genes with 7 NCG cancer genes. Especially, three genes TP53, MDM2 and TOPORS were identified for both ComCovEx and ComMDP with only TOPORS not being an NCG cancer gene. It indicates that TOPORS may be a potential cancer gene deserving further experimental verification.

#### 2.2.2 Breast Invasive Carcinoma (BRCA) and Head and Neck Squamous Cell Carcinoma (HNSC)

**Figure 6:**
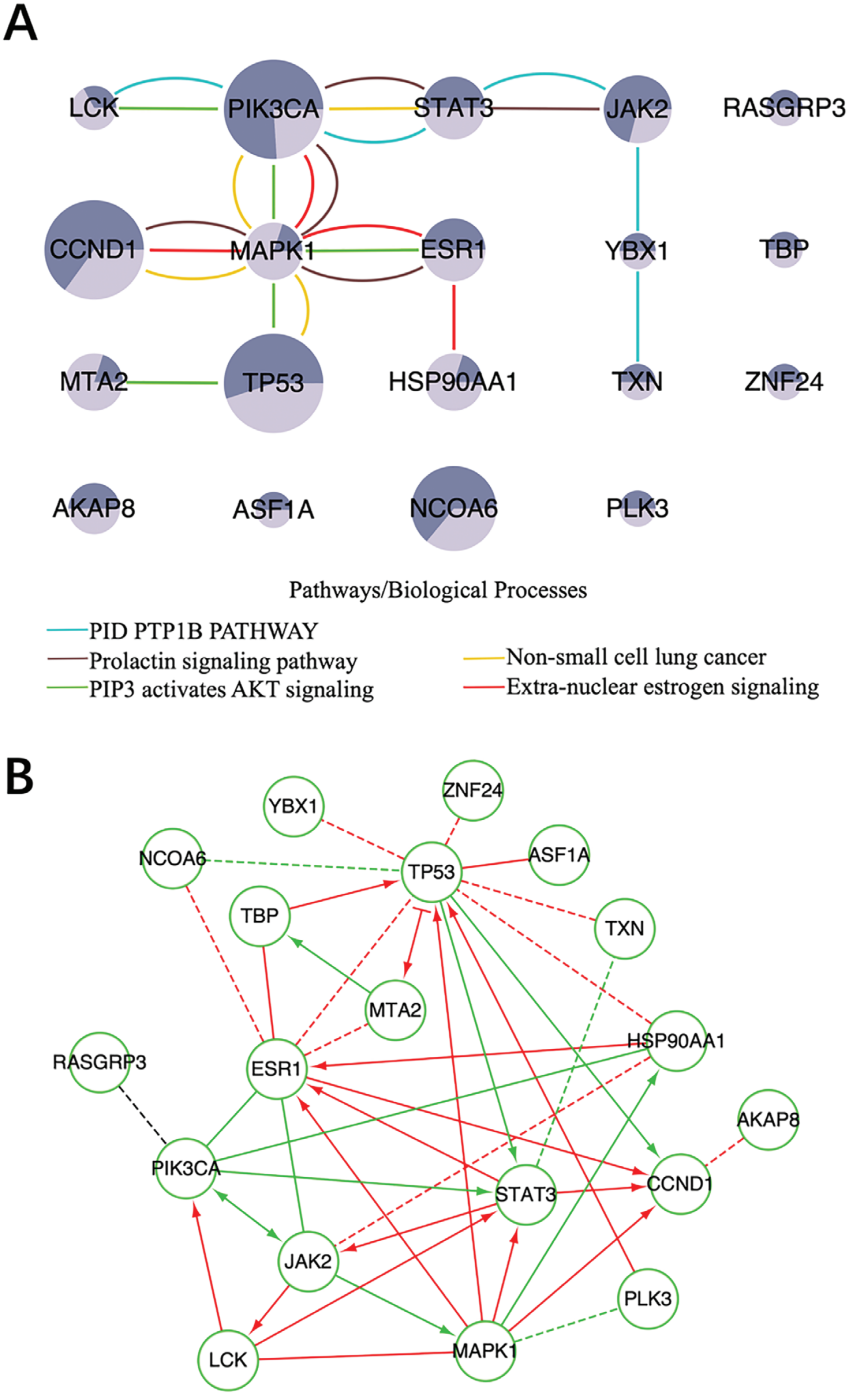
The enrichment analysis and the visualization of the driver gene module identified by ComCovEx for BRCA and HNSC. A) The enriched pathways and biological processes of the gene sets identified by ComCovEx for BRCA and HNSC. The meanings of the nodes and edges are the same as in Figure 5A. Especially, the two areas of dark and light colors at each gene are proportional to the coverages of the gene for BRCA and HNSC, respectively. B) The visualization of the induced network and the Reactome FI network of the identified driver genes for BRCA and HNSC. The induced network edges with paths in the Reactome FI network connecting the corresponding genes have been removed. The meanings of the edges are the same as in Figure 5B.

We identified a total of 19 driver genes with 11 NCG cancer genes in the filtered gene modules (**Figure** **6**A). The pathway and process enrichment analysis indicated that the enriched terms were grouped into eleven clusters (Figure S12, Supporting Information). The most significant five representative terms included one Canonical Pathways term, two KEGG Pathway terms and two Reactome Gene Sets terms, such as PID PTP1B PATHWAY (M50), Prolactin signaling pathway (hsa04917), Non‐small cell lung cancer (hsa05223), Extra‐nuclear estrogen signaling (R‐HSA‐9009391) and PIP3 activates AKT signaling (R‐HSA‐1257604).

Of all the 19 predicted driver genes, the most significant five representative terms covered 12 genes with PIK3CA, MAPK1, STAT3, CCND1 and ESR1 belonging to at least three representative terms. Especially, PIK3CA and MAPK1 belonged to five and four representative terms, respectively. All the significant eleven representative terms covered 16 genes with 7 genes CCND1, TP53, MAPK1, PIK3CA, JAK2, STAT3 and ESR1 belonging to at least four representative terms. In addition, four genes CCND1, TP53, MAPK1 and PIK3CA belonged to seven, six, five and five representative terms, respectively. Two genes AKAP8 and MTA2 belonged to three representative terms. The 16 genes covered by the representative terms included 11 NCG cancer genes with exception for MTA2, NCOA6, PLK3, TXN and YBX1. Three genes ASF1A, RASGRP3 and ZNF24 were not covered by any representative term and none of them is an NCG cancer gene.

Enrichment analysis in DisGeNET indicated that these genes were significantly enriched for adenocarcinoma (C0001418), mammary neoplasms (C1458155), squamous cell carcinoma (C0007137), leukemia (C0023418) and neoplasm metastasis (C0027627) (Figure S13, Supporting Information). Especially, seven genes CCND1, ESR1, HSP90AA1, YBX1, PIK3CA, STAT3, and TP53 were enriched for mammary neoplasms (C1458155). Five genes CCND1, PIK3CA, MAPK1, STAT3 and TP53 were enriched for squamous cell carcinoma (C0007137). Three genes PIK3CA, STAT3, and TP53 were enriched for squamous cell carcinoma of the head and neck (C1168401). Note that the enriched gene sets for all the three diseases included the same three genes PIK3CA, STAT3, and TP53.

We found a great many of overlaps between the induced network and the Reactome FI network of the identified driver genes (Figure S5, Supporting Information). After removing the induced network edges with paths in the Reactome FI network connecting the corresponding genes, only one induced network edge connecting PIK3CA and RASGRP3 left, indicting the possible unknown functional interaction (Figure 6B).

Combining all the identified gene sets with size from 2 to 10, ComMDP identified 12 cancer genes with 8 NCG cancer genes for BRCA and HNSC. Only Two NCG cancer genes TP53 and PIK3CA were identified for both ComCovEx and ComMDP.

#### 2.2.3 Breast Invasive Carcinoma (BRCA) and Lung Squamous Cell Carcinoma (LUSC)

**Figure 7:**
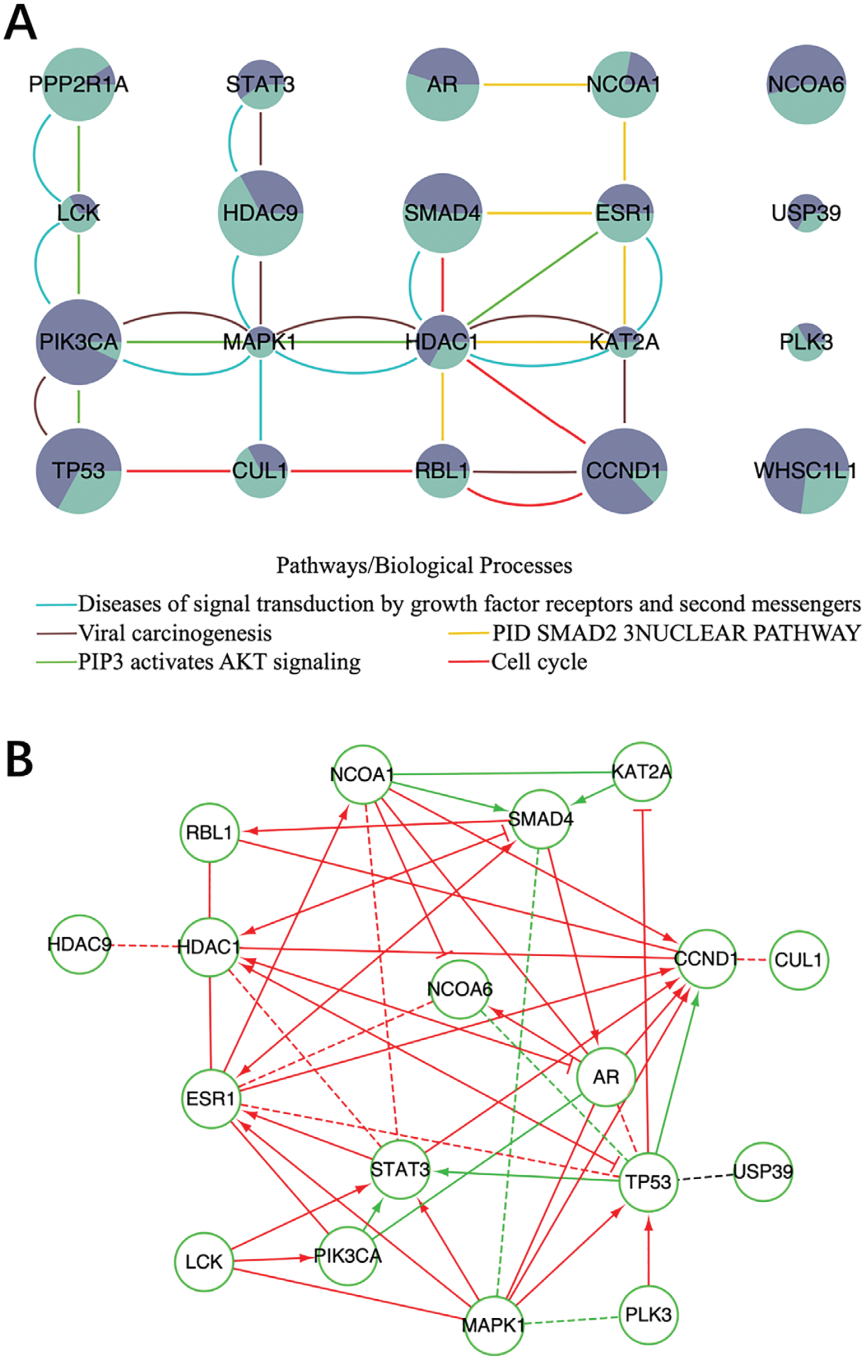
The enrichment analysis and the visualization of the driver gene module identified by ComCovEx for BRCA and LUSC. A) The enriched pathways and biological processes of the gene sets identified by ComCovEx for BRCA and LUSC. The meanings of the nodes and edges are the same as in Figure 5A. Especially, the two areas of dark and light green colors at each gene are proportional to the coverages of the gene for BRCA and LUSC, respectively. B) The visualization of the induced network and the Reactome FI network of the identified driver genes for BRCA and LUSC. The induced network edges with paths in the Reactome FI network connecting the corresponding genes have been removed. The meanings of the edges are the same as in Figure 5B.

We identified a total of 20 driver genes with 14 NCG cancer genes in the filtered gene modules (**Figure** **7**A). The pathway and process enrichment analysis indicated that the enriched terms were grouped into sixteen clusters (Figure S14, Supporting Information). The most significant five representative terms included two Reactome Gene Sets terms, two KEGG Pathway terms and one Canonical Pathways term, such as Diseases of signal transduction by growth factor receptors and second messengers (R‐HSA‐5663202), Viral carcinogenesis (hsa05203), PID SMAD2 3NUCLEAR PATHWAY (M2), Cell cycle (hsa04110) and PIP3 activates AKT signaling (R‐HSA‐1257604).

Of all the 20 predicted driver genes, the most significant five representative terms covered 16 genes with eight genes HDAC1, ESR1, KAT2A, SMAD4, PIK3CA, MAPK1, RBL1, and TP53 belonging to at least three representative terms. Especially, HDAC1 belonged to all the five representative terms. All the significant sixteen representative terms covered 18 genes with 15 genes belonging to at least four representative terms except for LCK, NCOA6, and WHSC1L1. In addition, four genes HDAC1, TP53, SMAD4, and MAPK1 belonged to eleven, eight, seven and seven representative terms, respectively. Another three genes CCND1, CUL1, and KAT2A belonged to six representative terms. The 18 genes covered by the representative terms included 14 NCG cancer genes with exception for HDAC1, KAT2A, NCOA6, and RBL1. Two genes PLK3 and USP39 were not covered by any representative term and none of them is an NCG cancer gene.

Enrichment analysis in DisGeNET indicated that these genes were significantly enriched for squamous cell carcinoma (C0007137), stomach neoplasms (C0038356), female infertility (C0021361), adenocarcinoma (C0001418) and mammary neoplasms (C1458155) (Figure S15, Supporting Information). Especially, seven genes AR, CCND1, ESR1, PIK3CA, STAT3, TP53, and NCOA1 were enriched for mammary neoplasms (C1458155). Five genes CCND1, ESR1, PIK3CA, MAPK1, and TP53 were enriched for lung neoplasms (C0024121). Clearly, four genes CCND1, ESR1, PIK3CA, and TP53 were related to both diseases. In addition, four genes CCND1, ESR1, STAT3, and NCOA1 were enriched for mammary neoplasms, experimental (C0024668).

We also found a great many of overlaps between the induced network and the Reactome FI network of the identified driver genes (Figure S6, Supporting Information). After removing the induced network edges with paths in the Reactome FI network connecting the corresponding genes, only one induced network edge connecting TP53 and USP39 left, indicting the possible unknown functional interaction (Figure 7B).

Combining all the identified gene sets with size from 2 to 10, ComMDP identified 13 cancer genes with 9 NCG cancer genes for BRCA and LUSC. Only Two NCG cancer genes TP53 and PIK3CA were identified for both ComCovEx and ComMDP.

#### 2.2.4 Significantly Associated Three‐Cancer Groups

For the 13 significantly associated cancer pairs, we identified 5 significantly associated three‐cancer groups within which each cancer pair was significantly associated (Figure 4). For the BRCA‐HNSC‐LUSC group, eight genes CCND1, ESR1, LCK, NCOA6, PIK3CA, PLK3, STAT3, and TP53 were identified for each cancer pair. Of the eight genes, six genes were NCG cancer genes except for NCOA6 and PLK3. Note that the six NCG cancer genes were all known cancer genes. The pathway and process enrichment analysis indicated that the enriched terms were grouped into five clusters (Figure S16, Supporting Information). The five representative terms included one KEGG Pathway term, one Reactome Gene Sets term and three GO Biological Processes terms, such as Non‐small cell lung cancer (hsa05223), Signaling by SCF‐KIT (R‐HSA‐1433557), mitotic G1 DNA damage checkpoint (GO:0031571), negative regulation of autophagy (GO:0010507) and positive regulation of proteolysis (GO:0045862). Enrichment analysis in DisGeNET also identified significantly enriched terms (Figure S17, Supporting Information). Four genes CCND1, PIK3CA, STAT3, and TP53 were enriched for Squamous cell carcinoma (C0007137). Three genes PIK3CA, STAT3, and TP53 were enriched for squamous cell carcinoma of the head and neck (C1168401). Five genes CCND1, ESR1, PIK3CA, STAT3, and TP53 were enriched for mammary neoplasms (C1458155). Four genes CCND1, ESR1, PIK3CA, and TP53 were enriched for lung neoplasms (C0024121). Three genes CCND1, ESR1, and STAT3 were enriched for mammary neoplasms, experimental (C0024668).

For the BRCA‐COADREAD‐UCEC group, five NCG cancer genes CTNNB1, MYC, PIK3CA, TLE1, and TP53 were identified for each cancer pair. The pathway and process enrichment analysis indicated that the enriched terms were grouped into six clusters (Figure S18, Supporting Information). The five representative terms included three KEGG Pathway terms and two GO Biological Processes terms, such as Endometrial cancer (hsa05213), fibroblast apoptotic process (GO:0044346), beta‐catenin‐TCF complex assembly (GO:1904837), Prostate cancer (hsa05215) and Signaling pathways regulating pluripotency of stem cells (hsa04550). Enrichment analysis in DisGeNET also identified significantly enriched terms (Figure S19, Supporting Information). Three genes CTNNB1, PIK3CA, and TP53 were enriched for leiomyosarcoma of uterus (C0280631), hereditary nonpolyposis colorectal carcinoma (C4024989) and breast carcinoma (C0678222).

For the BRCA‐COADREAD‐HNSC group, three genes PIK3CA, TP53, and ZNF24 were identified for each cancer pair with PIK3CA and TP53 being NCG and known cancer genes. For the COADREAD‐LUAD‐UCEC group, two NCG and known cancer genes KRAS and TP53 were identified for each cancer pair. For the COADREAD‐HNSC‐LUAD group, only TP53 was identified for each cancer pair.

## 3 Discussion

Identifying the common driver genes and modules between cancers is of great significance to understand the common pathogenic mechanism and provide new clues to diagnosis and drug treatment in related cancers. A simple approach for addressing the problem is to apply the driver gene/module identification methods for single cancer types directly to the merged dataset for multiple cancer types. Unfortunately, the properties of the identified genes/modules cannot be ensured for each cancer type in this case. Another idea for the problem is to take the intersection of the genes/modules identified by applying the single cancer type methods for different cancer types separately. However, important genes/modules that are not simultaneously optimal for all cancers will not be identified in this case. Therefore, novel methods are necessary for reasonably integrating multiple cancer datasets.

In fact, the current methods for processing different cancer datasets independently have not integrated multiple omics data and cannot make systematic searches.^[^ ^31^ ^]^ To tackle this problem, we designed ComCovEx by integrating protein‐protein interaction networks and multiple cancer datasets properly. The target of ComCovEx is to identify the topologically related gene modules with large coverages and mutually exclusive scores for multiple cancers, which are expected by an ideal common driver gene module for different cancers. By limiting the search space to numerous local influence networks, the genes in the identified gene modules would be topologically related. Meanwhile, we also achieved a large‐scale search for the target modules. By optimizing the minimum exclusive score of gene modules for different cancers and selecting gene modules with large coverages, the coverage and mutual exclusivity properties of the identified gene modules were also ensured for different cancers. The identified gene modules in all local influence networks were further evaluated and filtered by statistical analysis (see the Experimental Section).

The driver gene modules were identified by integrating the cancer genomic data with the influence network derived from a PPI network. The underlying PPI network is very important for the quality of the identified gene modules as well as the relationship between cancers. As shown in the paper, if restricting the identified gene modules to those genes significant for at least two PPI networks in our analysis, the prediction precision would be greatly improved. Intuitively, the improvement on accuracy and coverage of any single PPI network would also benefit the identification of driver gene modules.

The cancer genes and the induced network predicted by ComCovEx have been evaluated by using the NCG cancer genes and the Reactome FI network, respectively. Although the reliability of the predicted results has been verified to some extent, the functions of the predicted driver genes and interactions should be further investigated and confirmed by biological experiments. On the other hand, the predictions by the computational analysis would help prioritize the laboratory experiments. The limitation of ComCovEx is that it overemphasizes the mutual exclusivity rather than the coverage of the gene modules and the identified gene modules tend to be strictly exclusive in practice. Despite the importance of the strictly exclusive modules, new methods with better balance between coverage and mutual exclusivity may obtain new discoveries ignored by ComCovEx.

## 4 Conclusion

We presented a network‐based method ComCovEx to investigate the commonalities of driver gene modules and the associations between cancers. By applying to each pair of 11 cancer datasets, ComCovEx identified 13 significantly associated cancer pairs with plenty of biologically significant common gene modules and also 18 significantly unassociated cancer pairs. The identified common gene modules presented significant overlaps with known cancer genes and pathways in databases and literature and disclosed some possible unknown functional interactions deserved for further analysis and experimental investigation. The results of ComCovEx can be helpful for understanding the common pathological basis of different cancer types and provide new clues to diagnosis and drug treatment in associated cancers.

## 5 Experimental Section

### Datasets

The 11 TCGA cancer datasets covering 12 cancer types and the influence matrix files for three PPI networks HINT+HI2012, iRefIndex and Multinet were downloaded from http://compbio-research.cs.brown.edu/pancancer/hotnet2/.^[^ ^36^ ^]^ All the TCGA datasets contain 3106 samples with 11565 mutated genes after filtration with RNA‐seq expression data. Here, the mutated genes were defined as the genes with at least one mutation. The mutation data comprise somatic single‐nucleotide variants (SNVs), small indels and copy number aberrations (CNAs) including amplifications and deletions. The 11 datasets include bladder urothelial carcinoma (BLCA), breast invasive carcinoma (BRCA), colon adenocarcinoma and rectum adenocarcinoma (COADREAD), glioblastoma multiforme (GBM), head and neck squamous cell carcinoma (HNSC), kidney renal clear cell carcinoma (KIRC), acute myeloid leukemia (LAML), lung adenocarcinoma (LUAD), lung squamous cell carcinoma (LUSC), ovarian serous cystadenocarcinoma (OV) and uterine corpus endometrioid carcinoma (UCEC). Especially, the COADREAD dataset covers two cancer types and each other dataset covers only one cancer type. Each pair of datasets is shortly referred to as a cancer pair.

The influence matrix files for the three PPI networks were generated by HotNet2 through a random walk model with elements in each matrix as the influence scores evaluating the topological relationships of corresponding gene pairs in the PPI network.^[^ ^36^ ^]^ For each PPI network, an influence network of average node degree 15 was constructed with nodes corresponding to mutated genes and edges with large influence scores.

### Coverage and Mutual Exclusivity of Gene Modules

To identify candidate gene modules, the coverage and mutual exclusivity property of a gene module was evaluated. The coverage of a gene module was defined as the number of samples with mutated genes in the module. The exclusive score proposed by CovEx^[^ ^33^ ^]^ was employed to evaluate the exclusive degree of a gene module. The exclusive score of each gene in a given gene module was defined as the fraction of mutated exclusive patients to all mutated patients of the gene where the exclusive patients refer to patients with one and only one mutated gene in the gene module. The exclusive score of the gene module was defined as the average exclusive score of genes in the gene module.

### Discovery of Candidate Common Gene Modules

For two cancer types, first, the candidate common driver gene modules were identified. To improve the prediction accuracy, only those genes were considered that were mutated in at least two samples for each cancer type as the center genes. For each mutated center gene, a local influence network was extracted by starting from the gene and gradually exploring the neighbor genes until reaching the proper radius such that the size of the local network was less than a pre‐specified number (100 in the experiment). For each local influence network, candidate modules with an exclusivity‐coverage iteration strategy were identified which first selected the center gene, and then in each subsequent iteration, selected the gene that maximizes the minimum exclusive score of the two considered cancer types. If multiple genes meet the requirements simultaneously, we selected each of the genes that contributing to the largest coverage for any considered cancer type, respectively. Considering that the exclusivity property of a gene module tended to get worse as the module size increased, the iteration ended when the module size reached 5. Then the genes were output according to the iterative sequence such that the sub‐modules identified in pervious iterations could be analyzed. Here, the size of a module is the number of genes in the module. The greedy strategy is described in Algorithm 1–2 in Table S11 in the Supporting Information.

### Statistical Tests and Prediction of the Common Driver Gene Modules

The above strategy identified the candidate common gene modules for two cancer types in each of the local influence networks. For each module size ranging from 2 to 5, the module coverage and the module exclusive score distributions were obtained by randomly selecting 10 million connected sub‐networks of the same size from the influence network and calculating the coverages and the exclusive scores of the corresponding modules. For each cancer type, the significant modules were defined to be those with both the coverage *p*‐value and the exclusive score *p*‐value no more than 0.05 based on the module coverage and the module exclusive score distributions, respectively. The significant modules for both considered cancer types were predicted as the common driver gene modules.

### Association Analysis between Cancers

It was assumed that the number of the identified common driver gene modules should be large for the highly associated cancer pairs. For a given considered module size and a PPI network, the associations of a cancer pair were quantified by Fisher's exact test which calculated the significance of the number of the identified common driver gene modules relative to the numbers of the significant modules of each cancer type and the number of all candidate modules. In the application, different module sizes ranging from 2 to 5 were tested based on each of the three PPI networks. For each cancer pair, ComCovEx obtained twelve *p‐*values with Fisher's exact test.

It should be noted that there exists containment relationship between different size modules for the same PPI network. Therefore, the twelve *p*‐values for each cancer pair were not independent and cannot be adjusted or integrated directly. For each cancer pair, the quartiles of the twelve *p‐*values were determined to evaluate the association degree of a cancer pair. For each of the three quartiles, the corresponding 55 *p‐*values of the 55 cancer pairs were further adjusted using the Benjamini–Hochberg (BH) method^[^ ^68^ ^]^ with *p.adjust* function in R to control the false discovery rate (FDR).

For each cancer pair, if the upper quartile (Q3) value was significant, it could be concluded that at least 75% *p*‐values were significant. If the lower quartile (Q1) value was non‐significant, it could be concluded that at least 75% *p*‐values were non‐significant. It was assumed that most of the twelve *p‐*values should be significant for a highly associated cancer pair. The cancer pairs with significant adjusted Q3 (<0.05) values were identified as significantly associated cancer pairs and the cancer pairs with non‐significant adjusted Q1 (>0.05) values were identified as significantly unassociated cancer pairs.

### The Relative Association Degree of a Cancer Relative to All the Other Cancers

Based on the adjusted Q2 or Q3 values of all cancer pairs, two ways were considered to assign scores for cancer pairs which were denoted as the logarithm score and the rank score. For the logarithm way, a score of a cancer pair was assigned as the negative logarithm of the corresponding Q2 or Q3 value. For the rank way, the Q2 or Q3 values of all cancer pairs were sorted in descending order and assigned the score of a cancer pair as the rank minus one. For each cancer type, the logarithm/rank score was defined as the sum of all the logarithm/rank scores of the cancer pairs containing the caner type. Ideally, the higher the score of a cancer, the stronger the association degree of the cancer relative to the other cancers.

### Evaluation of the Performance of Methods

To evaluate the identified genes, 2372 NCG cancer genes were downloaded with 711 known cancer genes from The Network of Cancer Genes (NCG) 6.0 as the gold standard.^[^ ^69^ ^]^ The NCG 6.0 is a manually curated repository of 2372 genes whose somatic modifications have known or predicted cancer driver roles.^[^ ^69^ ^]^ The precision of NCG/known cancer genes for the predicted gene modules of ComCovEx was defined as the fraction of the identified NCG/known cancer genes to the union of the genes in the modules output by ComCovEx.

## Conflict of Interest

The authors declare no conflict of interest.

## Author Contributions

G.L. and L.‐Y.W. designed the project and supervised the experimental analysis. B.G. developed the software. B.G., Y.Z., and Y.G. analyzed the data and performed the experiments. G.L., L.‐Y.W., and B.G. wrote the manuscript. Y.Z. processed the Figures.

## Supporting information

Supporting Information

## Data Availability

Data sharing is not applicable to this article as no new data were created or analyzed in this study.
