# Supplementary material for: Identification of Common Driver Gene Modules and Associations between Cancers through Integrated Network Analysis
Source: Glob Chall. 2021 Jun 19;5(9):2100006. doi: 10.1002/gch2.202100006 (PMC8414517; doi:10.1002/gch2.202100006)
Supplement: Supplementary file 1 — Supporting Information [file GCH2-5-2100006-s001.pdf]

© 2021 Wiley-VCH GmbH

Global Challenges

Supporting Information

for *Global Challenges,* DOI: 10.1002/gch2.202100006

Identification of Common Driver Gene Modules and Associations between Cancers through Integrated Network Analysis

Bo Gao, Yue Zhao, Yonghang Gao, Guojun Li, and Ling-Yun Wu**

Supplemental Material

Table of Content

Supplementary Tables
Supplementary Figures

Supplementary Tables

Table S1. The PCC values of each cancer pair based on the results of a previous computational study (BMC Genomics 2015, 16:503).

	UCEC	COADREAD	LUAD	BRCA	OV	KIRC	LAML	HNSC	GBM	BLCA	LUSC
UCEC	1.00	**1.00**	**0.87**	**1.00**	0.78	-0.01	0.41	0.30	0.10	0.59	0.15
COADREAD	**1.00**	1.00	**0.87**	**1.00**	0.78	-0.01	0.41	0.30	0.10	0.59	0.15
LUAD	**0.87**	**0.87**	1.00	**0.87**	**0.80**	-0.18	0.22	0.41	0.23	0.71	0.20
BRCA	**1.00**	**1.00**	**0.87**	1.00	0.78	-0.01	0.41	0.30	0.10	0.59	0.15
OV	0.78	0.78	**0.80**	0.78	1.00	-0.15	0.13	0.27	-0.25	0.35	0.33
KIRC	-0.01	-0.01	-0.18	-0.01	-0.15	1.00	-0.16	-0.43	-0.22	-0.29	-0.32
LAML	0.41	0.41	0.22	0.41	0.13	-0.16	1.00	-0.26	-0.01	-0.07	-0.27
HNSC	0.30	0.30	0.41	0.30	0.27	-0.43	-0.26	1.00	0.67	**0.82**	0.69
GBM	0.10	0.10	0.23	0.10	-0.25	-0.22	-0.01	0.67	1.00	0.76	0.42
BLCA	0.59	0.59	0.71	0.59	0.35	-0.29	-0.07	**0.82**	0.76	1.00	0.47
LUSC	0.15	0.15	0.20	0.15	0.33	-0.32	-0.27	0.69	0.42	0.47	1.00

Table S2. The logarithm score for each cancer type by considering the adjusted Q2 values of all cancer pairs. The cancer types are ranked in descending order of the logarithm scores.

Cancer type	The logarithm score
HNSC	189.60

BRCA	180.39
COADREAD	148.35
LUSC	101.66
LUAD	97.39
BLCA	81.68
UCEC	43.59
GBM	37.08
KIRC	28.89
OV	0.07
LAML	0

Table S3. The rank score for each cancer type by considering the adjusted Q2 values of all cancer pairs. The cancer types are ranked in descending order of the rank scores.

Cancer type	The rank score
BRCA	345
HNSC	334
LUAD	318
COADREAD	279
LUSC	277
BLCA	275
UCEC	237
KIRC	221
GBM	196
OV	22
LAML	0

Table S4. The logarithm score for each cancer type by considering the adjusted Q3 values of all cancer pairs. The cancer types are ranked in descending order of the logarithm scores.

Cancer type	The logarithm score
HNSC	51.05
BRCA	46.62
COADREAD	37.63
LUSC	33.17
LUAD	28.06
UCEC	20.09
BLCA	18.94
KIRC	10.23
GBM	2.87
LAML	0
OV	0

Table S5. The rank score for each cancer type by considering the adjusted Q3 values of all cancer pairs. The cancer types are ranked in descending order of the rank scores.

Cancer type	The rank score
HNSC	320
BRCA	275
LUAD	258
COADREAD	231
LUSC	184
UCEC	174
BLCA	136
KIRC	123
GBM	79
LAML	0
OV	0

Table S6. The numbers of all output genes, NCG genes, known cancer genes and the corresponding cancer gene precision for the 13 significantly associated cancer pairs before the filtration.

Cancer pairs	Number of all output genes	Number of NCG genes	Precision of NCG genes	Number of known cancer genes	Precision of known cancer genes
BLCA_LUAD	36	18	50.0%	12	33.3%
BLCA_LUSC	42	17	40.5%	11	26.2%
BRCA_COADREAD	44	17	38.6%	12	27.3%
BRCA_HNSC	73	34	46.6%	18	24.7%
BRCA_LUSC	66	31	47.0%	22	33.3%
BRCA_UCEC	58	28	48.3%	12	20.7%
COADREAD_HNSC	24	11	45.8%	10	41.7%
COADREAD_LUAD	43	23	53.5%	15	34.9%
COADREAD_UCEC	39	22	56.4%	14	35.9%
HNSC_KIRC	82	44	53.7%	25	30.5%
HNSC_LUAD	49	23	46.9%	16	32.7%
HNSC_LUSC	113	47	41.6%	28	24.8%
LUAD_UCEC	38	17	44.7%	13	34.2%

Table S7. The numbers of output genes, NCG genes, known cancer genes and the corresponding cancer gene precision for the 13 significantly associated cancer pairs after the filtration.

Cancer pairs	Number of output genes	Number of NCG genes	Precision of NCG genes	Number of known cancer genes	Precision of known cancer genes

BLCA_LUAD	19	13	68.4%	10	52.6%
BLCA_LUSC	20	9	45.0%	6	30.0%
BRCA_COADREAD	17	10	58.8%	7	41.2%
BRCA_HNSC	19	11	57.9%	9	47.4%
BRCA_LUSC	20	14	70.0%	12	60.0%
BRCA_UCEC	19	10	52.6%	5	26.3%
COADREAD_HNSC	12	5	41.7%	5	41.7%
COADREAD_LUAD	20	11	55.0%	8	40.0%
COADREAD_UCEC	25	16	64.0%	12	48.0%
HNSC_KIRC	25	16	64.0%	11	44.0%
HNSC_LUAD	29	16	55.2%	11	37.9%
HNSC_LUSC	35	20	57.1%	16	45.7%
LUAD_UCEC	19	10	52.6%	8	42.1%

Table S8. The common gene sets identified by ComMDP for BLCA and LUAD.

K	Common gene set	p_1	p_2	p
2	KRAS TP53	0.0004	0	0
3	KRAS PALB2 TP53	0.0001	0	0
4	KRAS PALB2 RSRC2 TP53	0	0	0
5	ARID4A KRAS PALB2 RSRC2 TP53	0	0	0
6	ARID4A KRAS PALB2 RENBP RSRC2 TP53	0	0	0
7	KIAA1024 KRAS PALB2 RENBP RSRC2 TP53 USP15	0	0	0
8	AMIGO3 BMS1 KIAA1024 KRAS PALB2 RENBP RSRC2 TP53	0	0	0
9	CRIPAK LILRB2 MDM2 MICAL3 STS SZT2 TOPORS TP53 WRN	0	0	0
10	CRIPAK LILRB2 LRP12 MDM2 MICAL3 STS SZT2 TOPORS TP53 WRN	0	0	0

K denotes the number of genes in a gene set. p_1 and p_2 denote the *p*-values of the common gene sets in BLCA and LUAD, respectively. *p* represents the overall significance.

Table S9. The common gene sets identified by ComMDP for BRCA and HNSC.

K	Common gene set	p_1	p_2	p
2	PIK3CA TP53	0	0.0001	0
3	GATA3 PIK3CA TP53	0	0.0001	0
4	CDH1 GATA3 MAP3K1 TP53	0	0.0007	0
5	CDH1 GATA3 MAP2K4 MAP3K1 TP53	0	0.0007	0
6	CDH1 GATA3 MAP2K4 MAP3K1 NBEAL1 TP53	0	0.0003	0
7	CDH1 GATA3 MAP2K4 MAP3K1 NBEAL1 PLXNB3 TP53	0	0.0001	0
8	CDH1 GATA3 MAP2K4 MAP3K1 NBEAL1 PLXNB3 TP53 TRAPPC8	0	0.0001	0
9	ABCD1 CDH1 GATA3 MAP2K4 MAP3K1 NBEAL1 PLXNB3	0	0	0

	TP53 TRAPPC8			
10	CDH1 GATA3 HLA-B IKBKAP MAP2K4 MAP3K1 NBEAL1 PLXNB3 TP53 TRAPPC8	0	0	0

K denotes the number of genes in a gene set. p_1 and p_2 denote the *p*-values of the common gene sets in BRCA and HNSC, respectively. *p* represents the overall significance.

Table S10. The common gene sets identified by ComMDP for BRCA and LUSC.

K	Common gene set	p_1	p_2	*p*
2	PIK3CA TP53	0	0.0005	0
3	GATA3 PIK3CA TP53	0	0.0004	0
4	CACHD1 GATA3 PIK3CA TP53	0	0.0004	0
5	ARHGAP23 CACHD1 GATA3 PIK3CA TP53	0	0.0004	0
6	CDH1 CTCF GATA3 MAP2K4 MAP3K1 TP53	0	0.0009	0
7	CDH1 CRIPAK CTCF GATA3 MAP2K4 MAP3K1 TP53	0	0.0009	0
8	CDH1 CRIPAK CTCF GATA3 MAP2K4 MAP3K1 TP53 ZSWIM5	0	0	0
9	CDH1 CRIPAK CTCF DHX37 GATA3 MAP2K4 MAP3K1 TP53 ZSWIM5	0	0	0
10	CDH1 CRIPAK CTCF DHX37 GATA3 MAP2K4 MAP3K1 MCC TP53 ZSWIM5	0	0	0

K denotes the number of genes in a gene set. p_1 and p_2 denote the *p*-values of the common gene sets in BRCA and LUSC, respectively. *p* represents the overall significance.

Table S11. The greedy strategy for the discovery of candidate common gene modules between cancers.

Algorithm 1. Identifying candidate gene modules in each local network

Input: mutation matrices A, B; influence network I; constant positive integer *k*

Output: the candidate common driver gene modules

Set *solutions* ←∅;

foreach considered center gene g_c **do**

solutions ← *solutions* ∪ GETSOLUTIONS(G, g_c);

G is the local network centered at g_c.

end

Return *solutions.*

Algorithm 2. GETSOLUTIONS

Input: set *G* of genes in the local networks; current gene module (solution) *M*

Output: the candidate common driver gene modules containing *M*

* $Cov_1(M)$, $Cov_2(M)$, $Ex_1(M)$, $Ex_2(M)$ is the coverage and the exclusive score of the gene module M for the two considered cancer types, respectively. For a gene g, $Ex(M \cup 4.4.)=\min\{Ex_1(M\cup\{g\}5), Ex_2(M\cup 5)\}$. *

If |M|=k then

  Return M;

end

$nextGenes \leftarrow \varnothing$;

If only one gene g maximizes $Ex(M\cup\{g\})$ then

  $nextGenes \leftarrow nextGenes \cup \{g\}$;

Else If each gene g in S=5(s>1) maximizes $Ex(M\cup\{g\})$ then

  foreach $g \in S$ do

    If $Cov_1(M\cup\{g\})$ OR $Cov_2(M\cup\{g\})$ is maximized for $g \in S$ then

      $nextGenes \leftarrow nextGenes \cup \{g\}$;

    end

  end

end

$newSols \leftarrow \varnothing$;

foreach g$\in$ $nextGenes$ do $newSols \leftarrow newSols \cup$ GETSOLUTIONS(G, $M\cup\{g\}$).

Return *newSols.*

Supplementary Figures

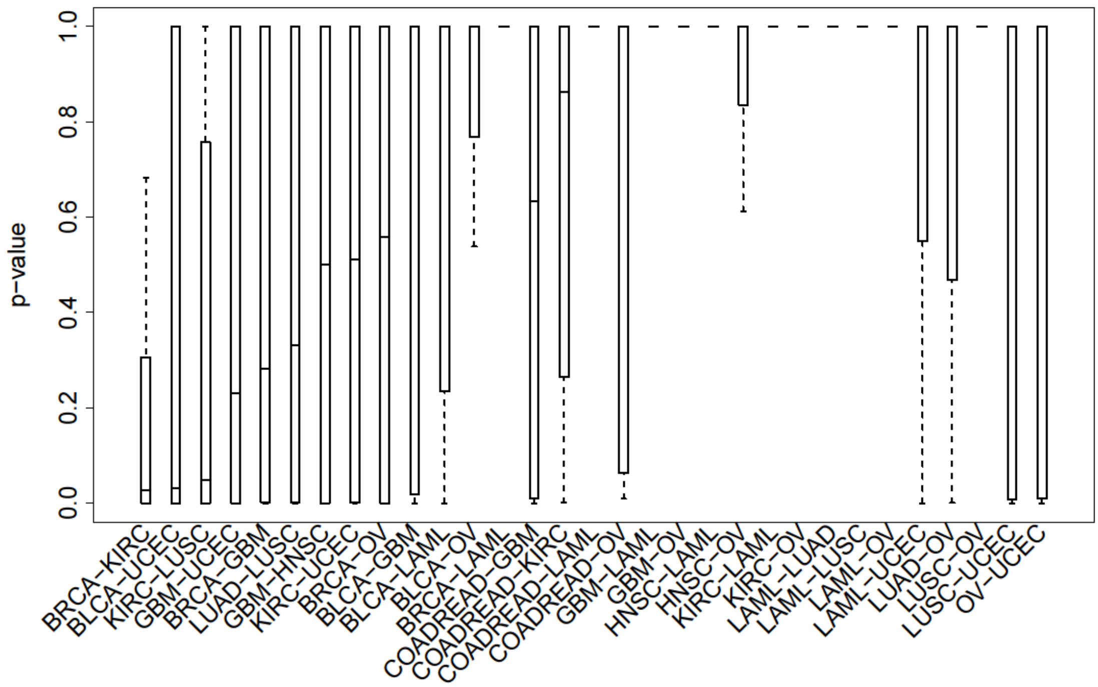

Figure S1. The boxplot of the *p*-values of the 31 cancer pairs with non-significant adjusted median (> 0.05) *p*-values. The sequence of the cancer pairs is arranged by the adjusted median *p*-values in an ascending order.

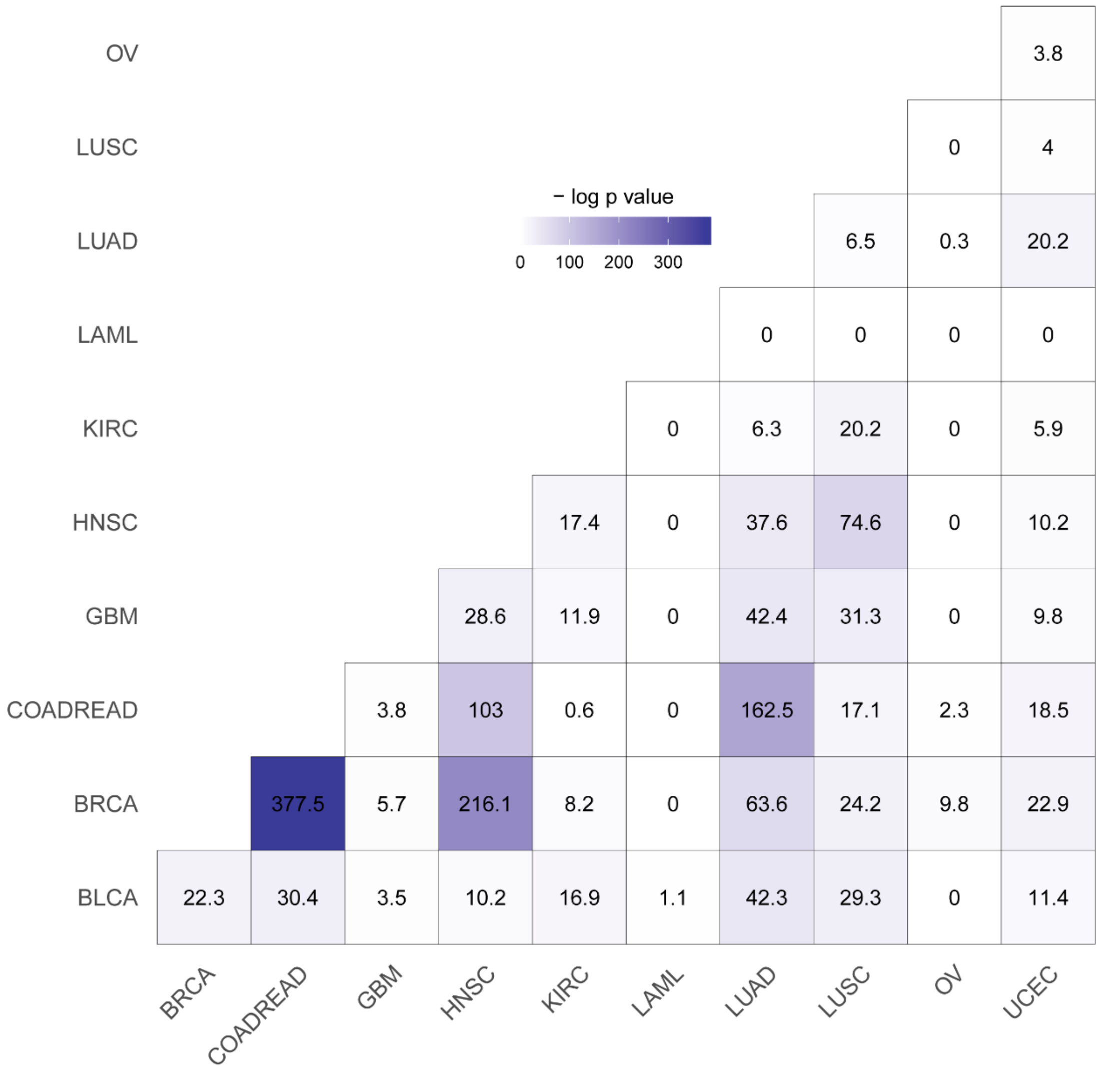

Figure S2. The negative logarithms (base e) of the adjusted Q1 values of all the 55 cancer pairs. Of all cancer pairs, we had 37 cancer pairs with significant adjusted Q1 (< 0.05) values. The negative logarithm values are at least 3.0 for the significant cancer pairs in this plot.

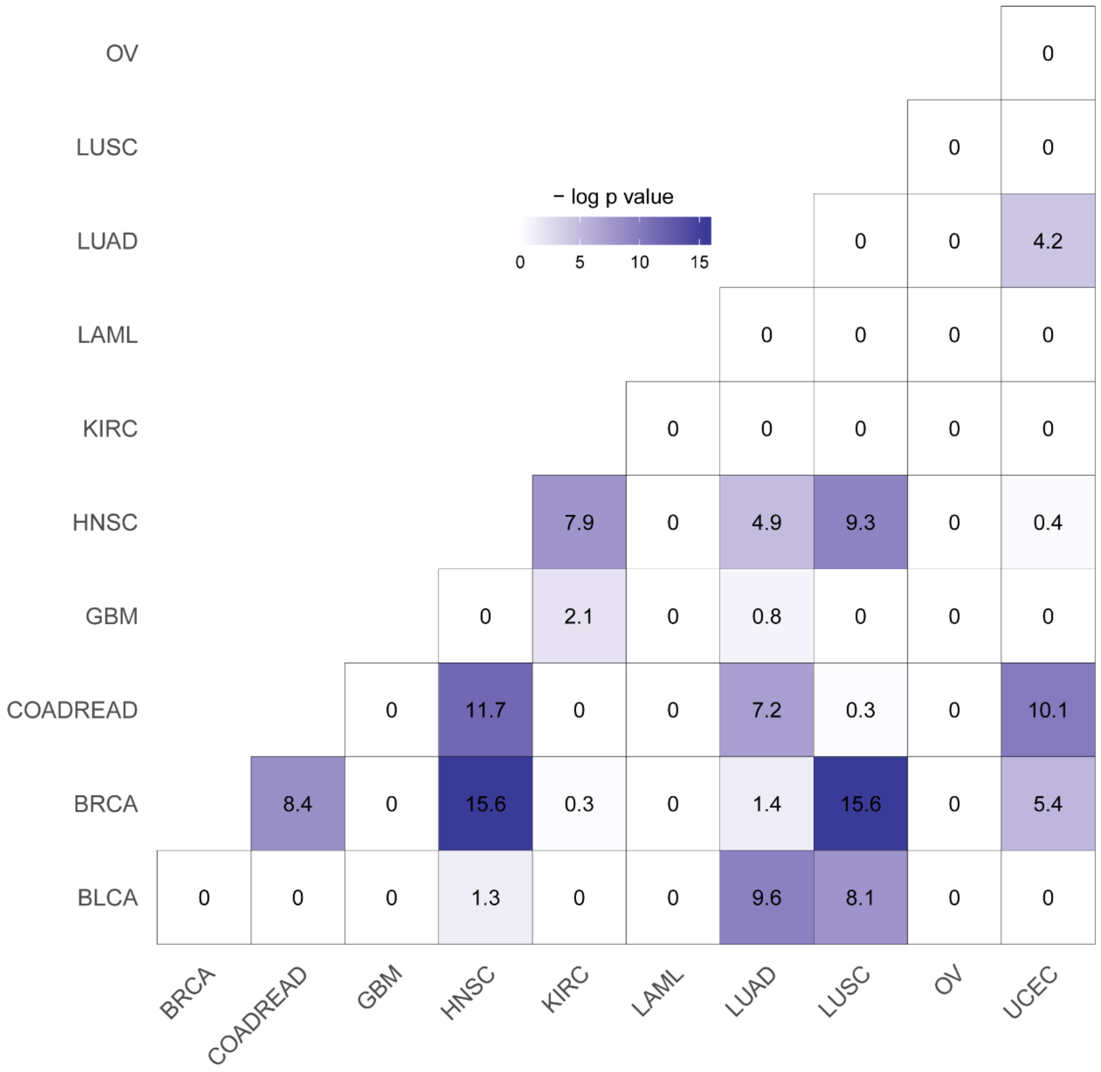

Figure S3. The negative logarithms (base e) of the adjusted Q3 values of all the 55 cancer pairs. Of all cancer pairs, we had 13 cancer pairs with significant adjusted Q3 (< 0.05) values. The negative logarithm values are at least 3.0 for the significant cancer pairs in this plot.

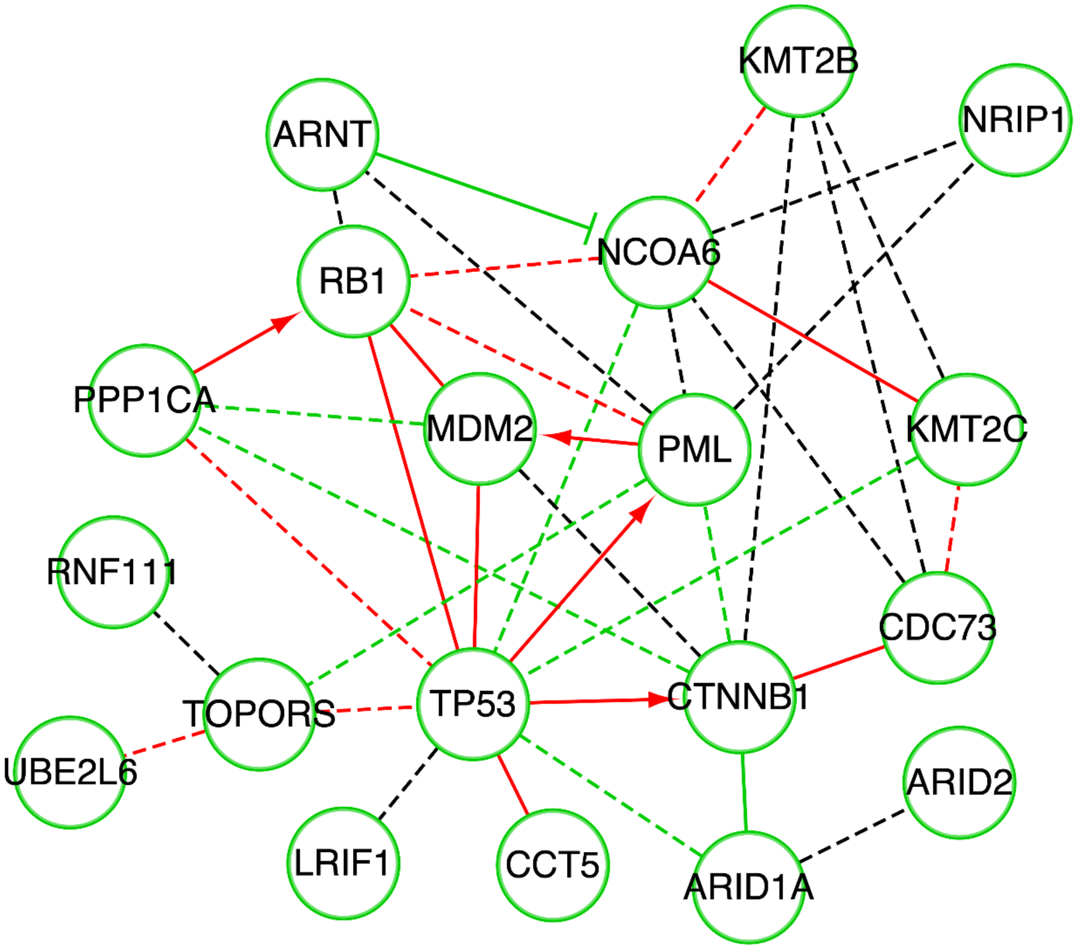

Figure S4. The visualization of the induced network and the Reactome FI network of the identified driver genes for BLCA and LUAD. The black dotted edges belong to the induced network and may reflect the possible indirect functional interactions between genes/proteins. Besides the black dotted edges, the other green and red edges are Reactome FI edges obtained directly from the plugin. According to the annotations of the plugin, "->" for activating/catalyzing, "-|" for inhibition, "-" for FIs extracted from complexes or inputs, and "---" for predicted FIs. Especially, the red edges are both the Reactome FI edges and the induced network edges.

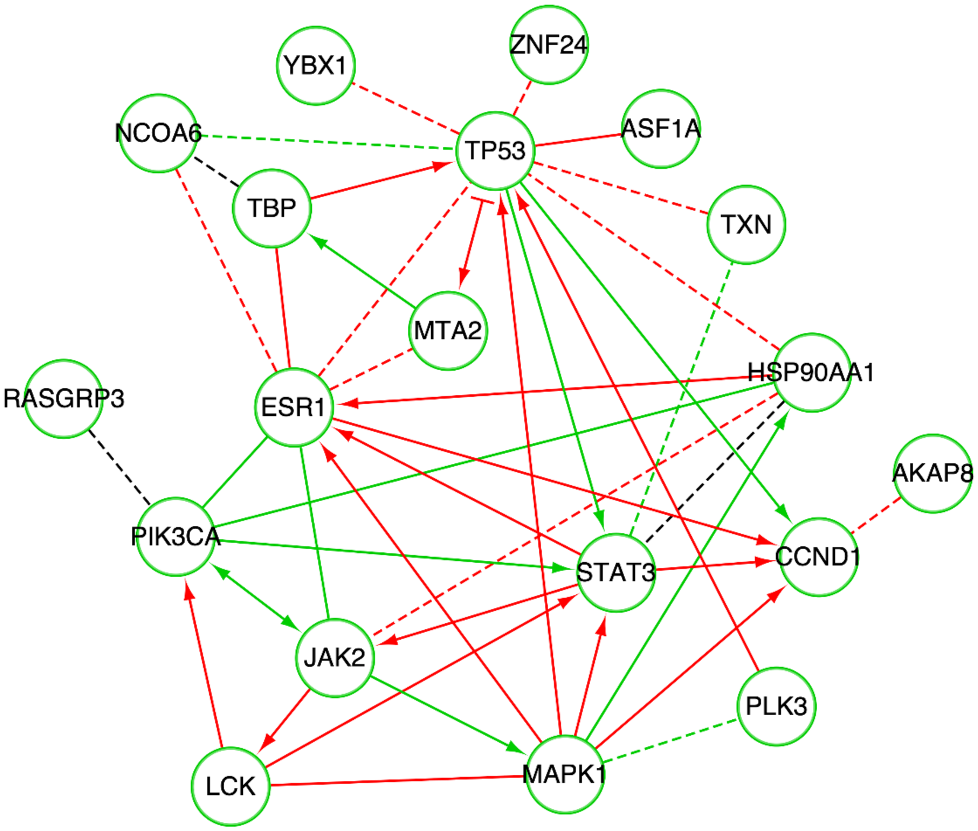

Figure S5. The visualization of the induced network and the Reactome FI network of the identified driver genes for BRCA and HNSC. The meanings of the edges are the same as in Figure S4.

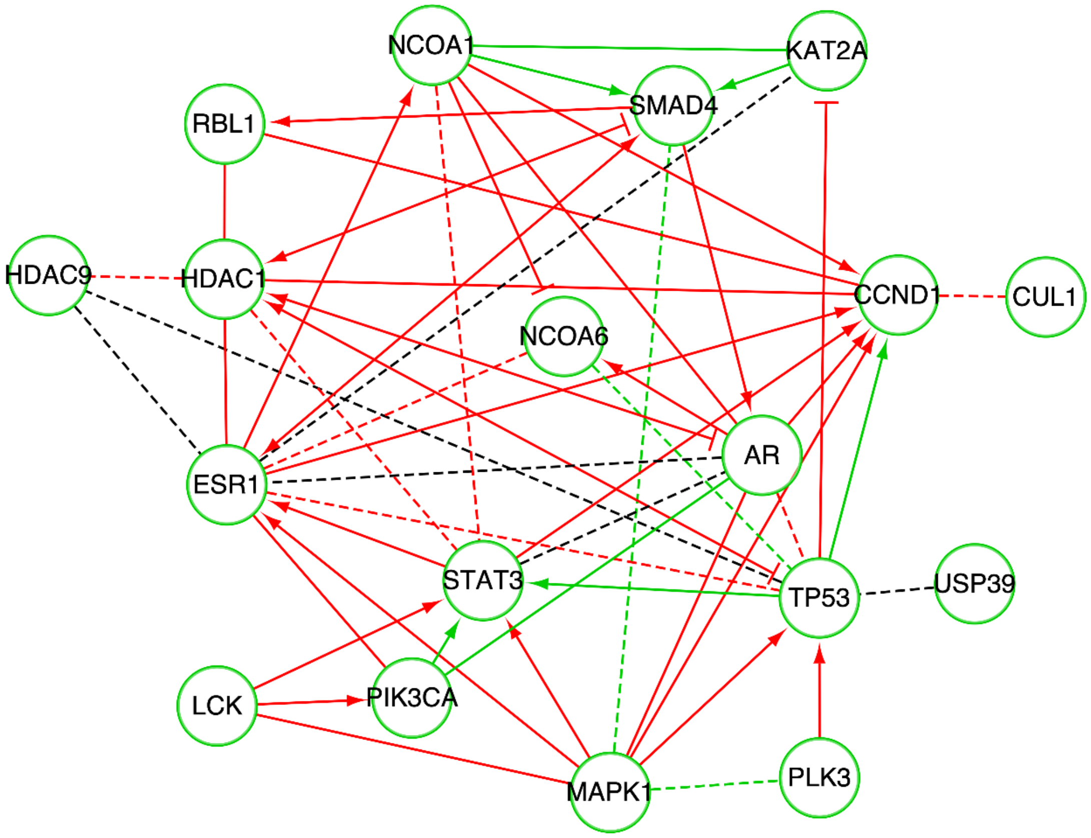

Figure S6. The visualization of the induced network and the Reactome FI network of the identified driver genes for BRCA and LUSC. The meanings of the edges are the same as in Figure S4.

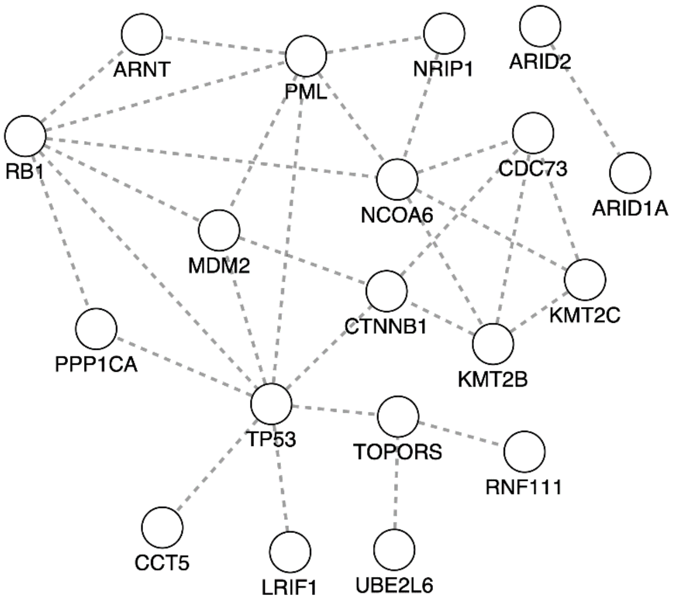

Figure S7. The visualization of the induced network and the known pathway network of the identified driver genes for BLCA and LUAD by GeneMANIA. The dotted edges belong to the induced network and may reflect the possible indirect pathway interactions between genes/proteins. The solid gray edges belong to the known pathway network. The red edges belong to both the induced network and the known pathway network indicting both the pathway and the strong topological relationship between the corresponding genes/proteins. Here, there is no known pathway network. In the induced network, TP53 has the maximum number of edges, followed by NCOA6, PML and RB1, indicting their important roles in PPI networks.

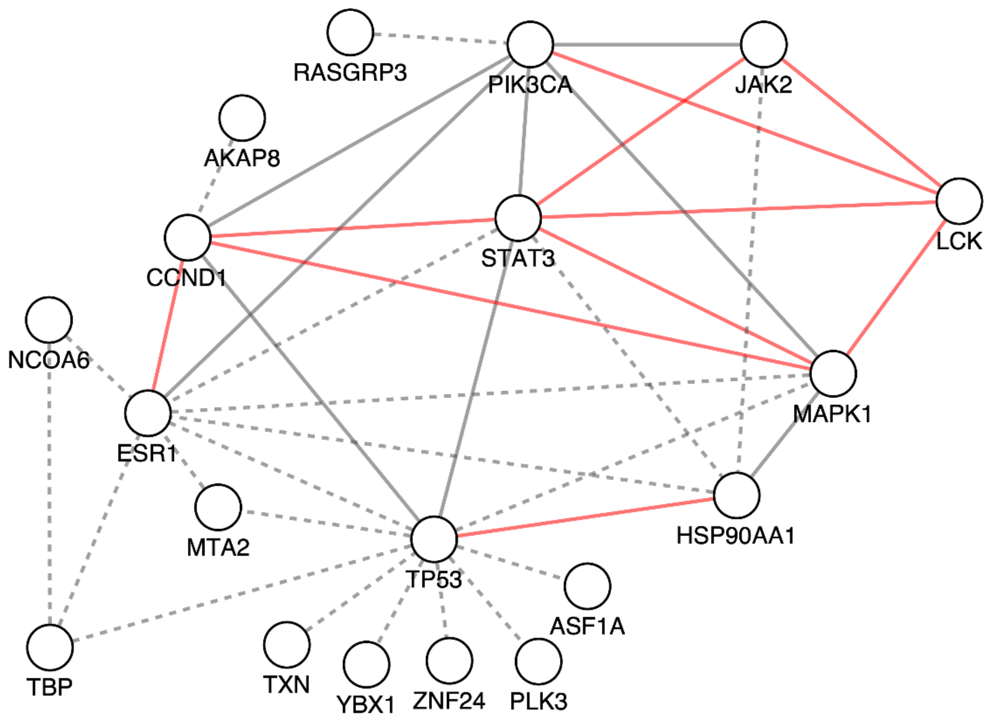

Figure S8. The visualization of the induced network and the known pathway network of the identified driver genes for BRCA and HNSC by GeneMANIA. The meanings of the edges are the same as in Figure S7. In the induced network, TP53 has the maximum number of edges, followed by ESR1, STAT3 and MAPK1.

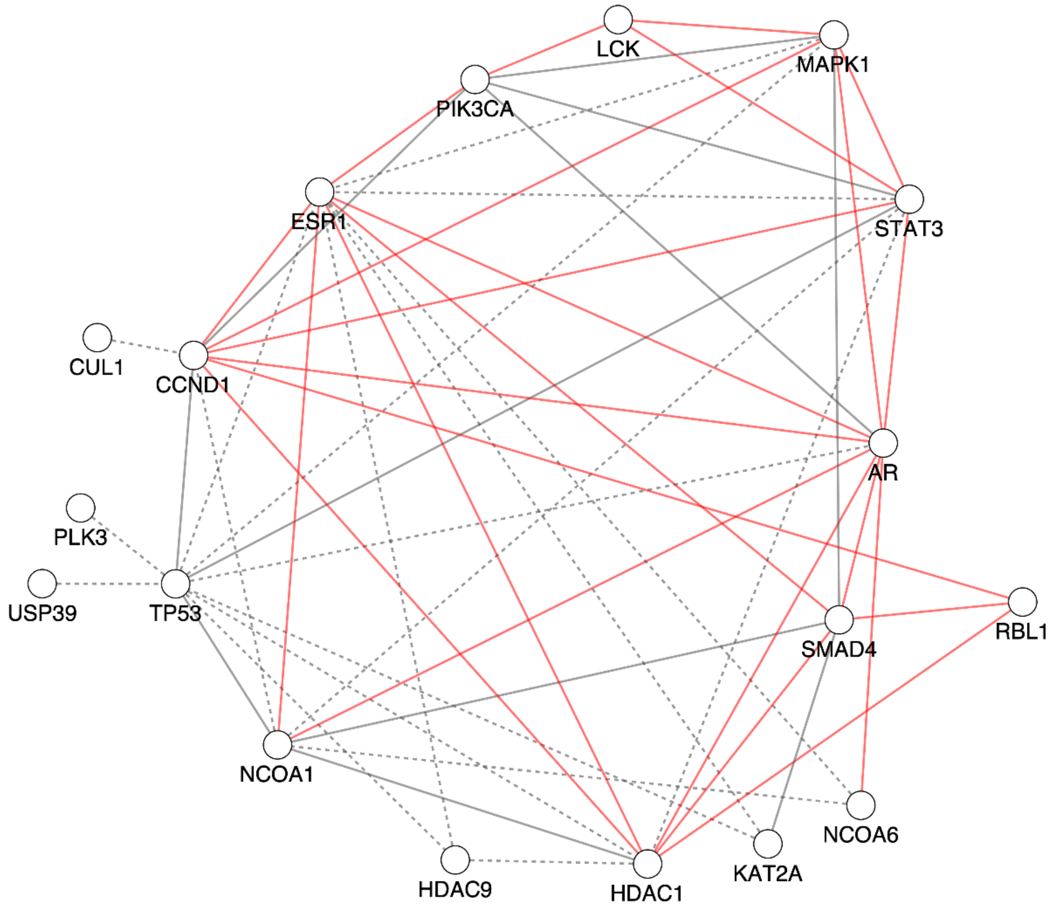

Figure S9. The visualization of the induced network and the known pathway network of the identified driver genes for BRCA and LUSC by GeneMANIA. The meanings of the edges are the same as in Figure S7. In the induced network, ESR1 has the maximum number of edges, followed by AR, CCND1, HDAC1 and TP53.

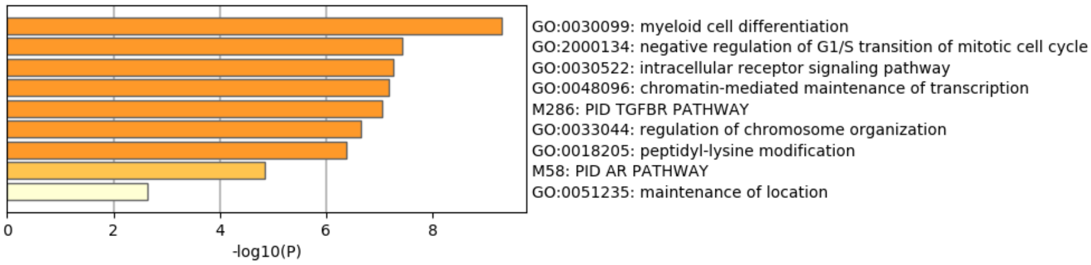

Figure S10. The bar graph of enriched terms identified by the pathway and biological process enrichment analysis across the identified gene set for BLCA and LUAD, colored by p-values.

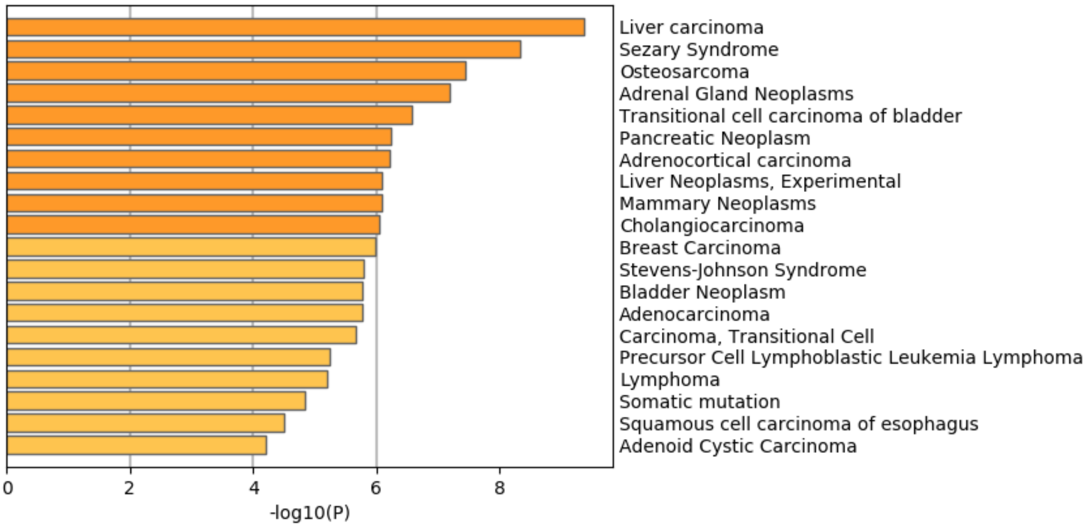

Figure S11. The bar graph of enriched terms identified by the enrichment analysis in DisGeNET across the identified gene set for BLCA and LUAD, colored by p-values.

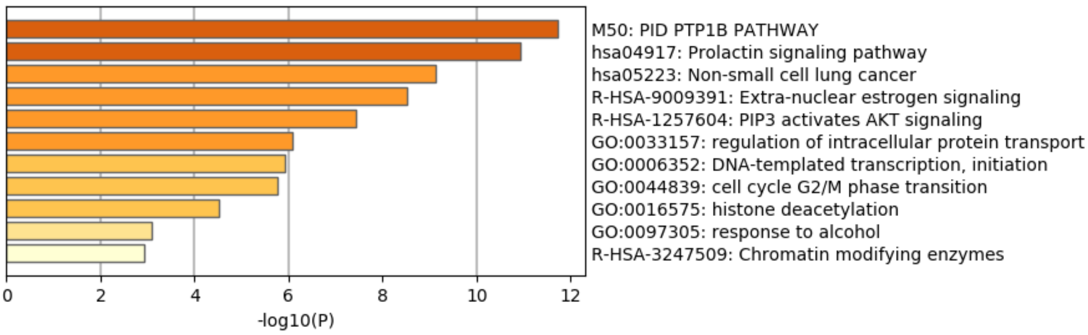

Figure S12. The bar graph of enriched terms identified by the pathway and biological process enrichment analysis across the identified gene set for BRCA and HNSC, colored by p-values.

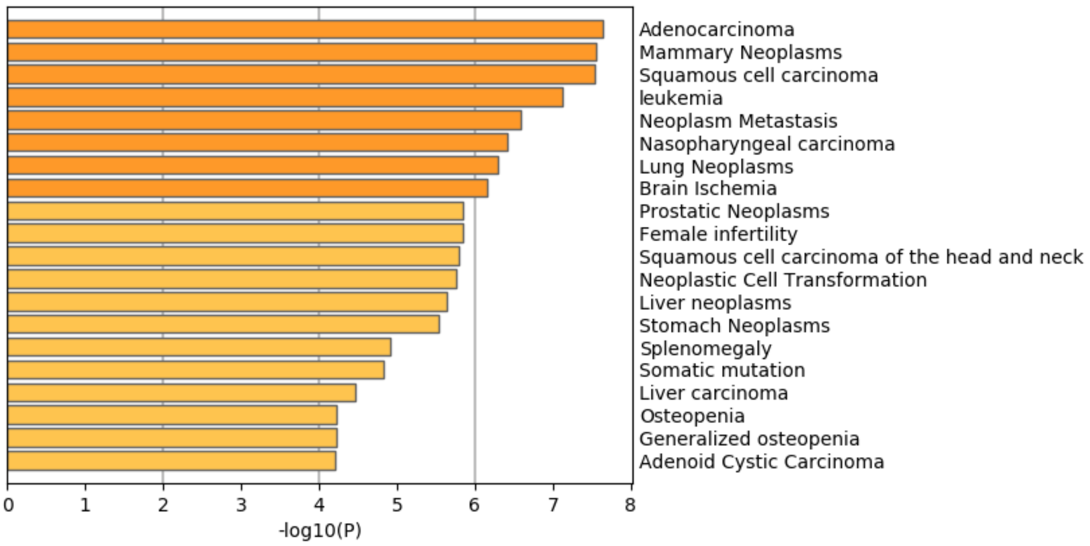

Figure S13. The bar graph of enriched terms identified by the enrichment analysis in DisGeNET across the identified gene set for BRCA and HNSC, colored by p-values.

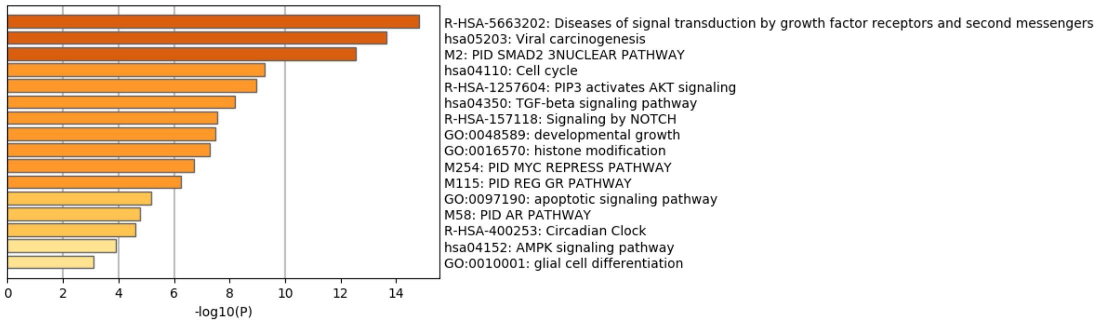

Figure S14. The bar graph of enriched terms identified by the pathway and biological process enrichment analysis across the identified gene set for BRCA and LUSC, colored by p-values.

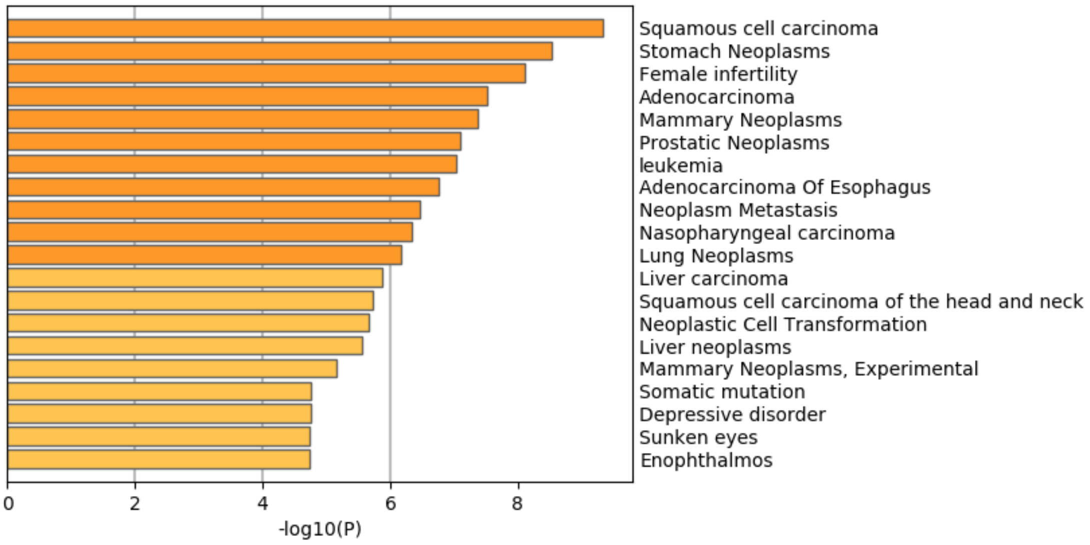

Figure S15. The bar graph of enriched terms identified by the enrichment analysis in DisGeNET across the identified gene set for BRCA and LUSC, colored by p-values.

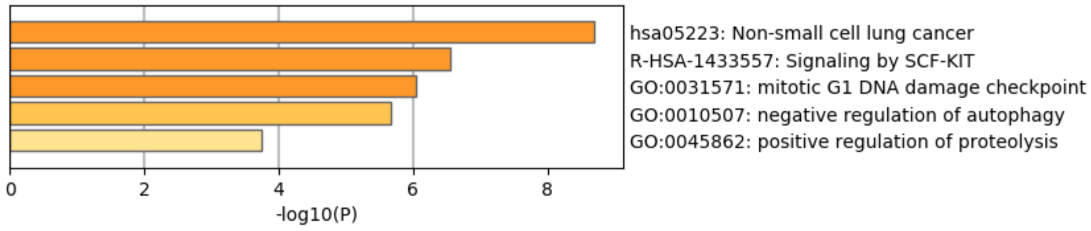

Figure S16. The bar graph of enriched terms identified by the pathway and biological process enrichment analysis across the common gene set for each cancer pair of the BRCA, HNSC and LUSC group, colored by p-values.

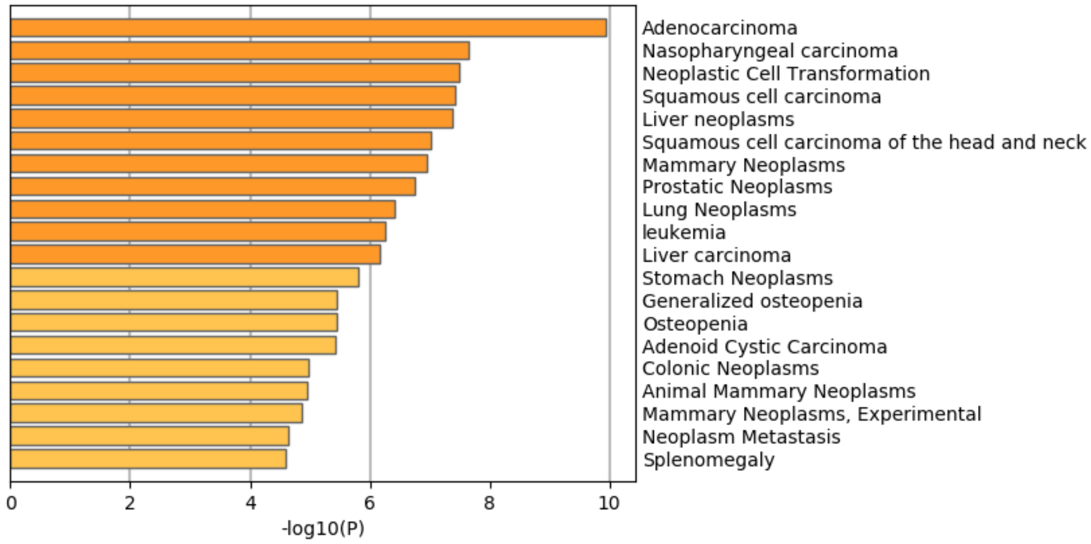

Figure S17. The bar graph of enriched terms identified by the enrichment analysis in DisGeNET across the common gene set for each cancer pair of the BRCA, HNSC and LUSC group, colored by p-values.

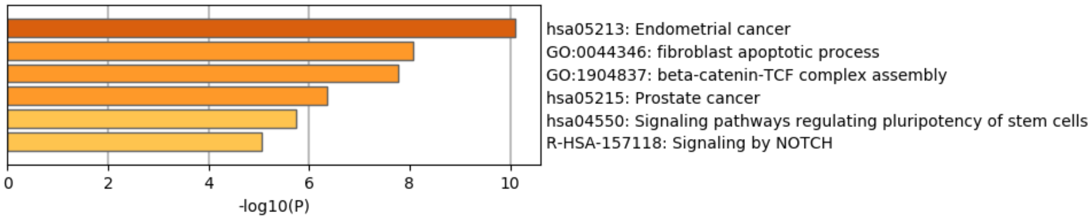

Figure S18. The bar graph of enriched terms identified by the pathway and biological process enrichment analysis across the common gene set for each cancer pair of the BRCA, COADREAD and UCEC group, colored by p-values.

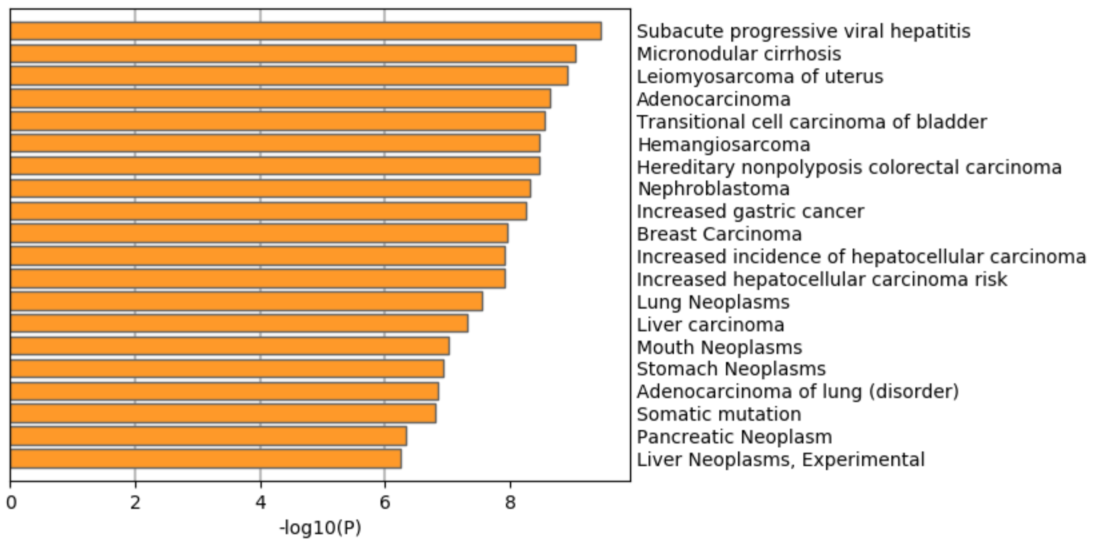

Figure S19. The bar graph of enriched terms identified by the enrichment analysis in DisGeNET across the common gene set for each cancer pair of the BRCA, COADREAD and UCEC group, colored by p-values.
